# The Coordinated Positive Regulation of Topoisomerase Genes Maintains Topological Homeostasis in Streptomyces coelicolor

**DOI:** 10.1128/JB.00530-16

**Published:** 2016-10-07

**Authors:** Marcin Jan Szafran, Martyna Gongerowska, Paweł Gutkowski, Jolanta Zakrzewska-Czerwińska, Dagmara Jakimowicz

**Affiliations:** a University of Wroclaw, Faculty of Biotechnology, Laboratory of Molecular Microbiology, Wroclaw, Poland; b Institute of Immunology and Experimental Therapy, Laboratory of Microbiology, Wroclaw, Poland; c Wroclaw University of Technology, Faculty of Chemistry, Wroclaw, Poland; Philipps-Universität Marburg

## Abstract

Maintaining an optimal level of chromosomal supercoiling is critical for the progression of DNA replication and transcription. Moreover, changes in global supercoiling affect the expression of a large number of genes and play a fundamental role in adapting to stress. Topoisomerase I (TopA) and gyrase are key players in the regulation of bacterial chromosomal topology through their respective abilities to relax and compact DNA. Soil bacteria such as Streptomyces species, which grow as branched, multigenomic hyphae, are subject to environmental stresses that are associated with changes in chromosomal topology. The topological fluctuations modulate the transcriptional activity of a large number of genes and in Streptomyces are related to the production of antibiotics. To better understand the regulation of topological homeostasis in Streptomyces coelicolor, we investigated the interplay between the activities of the topoisomerase-encoding genes *topA* and *gyrBA*. We show that the expression of both genes is supercoiling sensitive. Remarkably, increased chromosomal supercoiling induces the *topA* promoter but only slightly influences *gyrBA* transcription, while DNA relaxation affects the *topA* promoter only marginally but strongly activates the *gyrBA* operon. Moreover, we showed that exposure to elevated temperatures induces rapid relaxation, which results in changes in the levels of both topoisomerases. We therefore propose a unique mechanism of S. coelicolor chromosomal topology maintenance based on the supercoiling-dependent stimulation, rather than repression, of the transcription of both topoisomerase genes. These findings provide important insight into the maintenance of topological homeostasis in an industrially important antibiotic producer.

**IMPORTANCE** We describe the unique regulation of genes encoding two topoisomerases, topoisomerase I (TopA) and gyrase, in a model Streptomyces species. Our studies demonstrate the coordination of topoisomerase gene regulation, which is crucial for maintenance of topological homeostasis. Streptomyces species are producers of a plethora of biologically active secondary metabolites, including antibiotics, antitumor agents, and immunosuppressants. The significant regulatory factor controlling the secondary metabolism is the global chromosomal topology. Thus, the investigation of chromosomal topology homeostasis in Streptomyces strains is crucial for their use in industrial applications as producers of secondary metabolites.

## INTRODUCTION

Despite lacking a nucleus and a mitotic cell cycle, bacteria nevertheless must compact and organize their chromosomal DNA in a small cell volume. The first step of DNA compaction is its negative supercoiling (1–3), while further organization of the chromosome, referred to as a nucleoid, is aided by its association with nucleoid-associated proteins (NAPs) (4) and condensins (SMC proteins) (5). An accurate chromosomal spatial arrangement and the accumulation of negative supercoils promote DNA melting, which is crucial for the initiation of DNA replication and transcription (6–9). Thus, by decreasing the DNA melting energy and modifying the binding affinity of transcription regulators and/or sigma factors, supercoiling alterations serve as potent regulators of the selective expression of genes (10, 11). On the other hand, the movement of polymerase complexes along DNA during replication and transcription generates an excess of supercoils, which can inhibit both processes and consequently can be detrimental to cell growth (12). Moreover, chromosomal topology is affected by environmental conditions, such as heat shock. Interestingly, observations on the effects of elevated temperature on chromosome compaction were inconsistent, as either increased or decreased supercoiling could be detected in the analyzed bacterial species (13). Nevertheless, changes in DNA supercoiling are regarded as a regulatory mechanism that modulates transcription, thereby allowing the cell to adapt to environmental stress. Although the influence of DNA topology on global gene activity has been described, the responses of particular genes to changes in supercoiling may be directed by different mechanisms and are not fully understood.

In all cells, negative DNA supercoiling is maintained by the concerted action of a group of enzymes, called topoisomerases, that catalyze transient breaking and rejoining of phosphodiester bonds (14). Although the topoisomerase content varies in bacteria, two enzymes with opposing activities—topoisomerase I (TopA) and gyrase—are present in all known prokaryotes and comprise the minimal topology maintenance machinery. While topoisomerase I (a type I topoisomerase) removes negative supercoils by using free energy accumulated in the supercoiled DNA molecule, gyrase (a type II topoisomerase) introduces negative supercoils in an ATP-dependent manner (15). Thus, the opposing activities of these proteins result in the maintenance of global DNA supercoiling homeostasis. However, it remains unknown how the activities of these enzymes are coordinated.

The activities of TopA and gyrase are regulated in bacterial cells in several ways, i.e., by altering the availability of ATP (16), through direct interactions with the RNA polymerase (17), or by the binding of NAPs or Ssb proteins to DNA; the main regulatory mechanism is the control of transcription of the *topA* and *gyrAB* genes (11, 18, 19). Transcription of *topA* is controlled by either a single promoter (e.g., in Helicobacter pylori [20]) or multiple promoters (e.g., two promoters in Mycobacterium smegmatis and Mycobacterium tuberculosis [19] and four promoters in Escherichia coli [21]). The four E. coli *topA* promoters are controlled by different sigma factors, including the primary sigma factor (RpoD) and the heat shock sigma factor (RpoH) (21, 22). Thus, the level of *topA* transcription can be altered in response to a number of environmental signals (e.g., temperature up- and downshifts as well as osmotic and oxygen stresses) and other growth conditions (e.g., growth phase or host invasion) (19, 21, 23–25). In contrast to the TopA protein, which acts as a monomer, gyrase is composed of the subunits GyrA and GyrB, which are encoded by the *gyrA* and *gyrB* genes, respectively. In E. coli, both subunits are encoded and expressed independently (26, 27), whereas in Mycobacterium smegmatis, the *gyrA* and *gyrB* genes are organized in a single *gyrBA* operon and are driven from a single promoter (28). Studies of E. coli showed that in response to chromosomal relaxation, the *topA* promoter is remarkably downregulated (18), while the transcription of the gyrase genes is upregulated (28, 29). Moreover, in most bacteria, gyrase transcription is regulated by negative-feedback silencing, i.e., it is repressed by increased negative supercoiling, while *topA* gene transcription is only slightly stimulated under such conditions. The sensitivity of bacterial promoters to supercoiling conditions is correlated with the distribution of promoter elements, particularly the −10 and −35 regions as well as the spacer region, which is shorter in the *topA* promoter (13 to 16 bp) than the optimal length (17 bp) for the typical bacterial promoter (18, 19, 28, 30). Although the regulation of topoisomerase activity has been studied in model bacterial species, such as E. coli and M. smegmatis, little is known about the mechanisms maintaining topological homeostasis in soil bacteria, such as Streptomyces species, which are exposed to a number of environmental stresses.

Streptomyces strains are appreciated as producers of many useful secondary metabolites (i.e., antibiotics, immunosuppressants, and anticancer compounds), whose synthesis is associated with alterations of chromosomal topology (31, 32). Interestingly, the Streptomyces chromosome is a linear molecule that is maintained in circular form through the interaction of terminus-associated proteins (33). Streptomyces organisms resemble filamentous fungi in their mode of growth, which includes phases of vegetative growth and sporulation. During vegetative growth, the bacteria form a branched network of hyphae composed of elongated multigenomic cells which do not undergo typical division (34, 35). The multiple chromosomes present in hyphal cells are uncondensed and unsegregated. Environmental stress, particularly nutrient depletion, leads to morphological differentiation and the production of chains of unigenomic spores (35). This growth transition is accompanied by rapid changes in chromosomal topology, which allow the compaction of DNA into spores. Streptomyces coelicolor, a model Streptomyces species, similarly to other Actinobacteria, including M. smegmatis and M. tuberculosis, possesses only one gene encoding a type I topoisomerase (TopA). S. coelicolor TopA differs markedly from its bacterial homologues (with the exception of mycobacterial TopA) (36) in its high processivity and its atypical C-terminal domain, which lacks Zn fingers (37). This observation suggests that the removal of negative supercoils in S. coelicolor relies mostly on the activity of TopA, which must be regulated precisely to maintain the proper level of chromosomal supercoiling. It has also been shown for S. coelicolor that depletion of the TopA protein inhibits chromosomal segregation and multiple-cell division during sporulation (32). The maintenance of multiple-chromosome topology in Streptomyces species appears to be a challenging task, but little is known about the regulation of the TopA level in these bacteria and its coordination with gyrase activity.

In model bacteria, it has been shown that DNA supercoiling affects the transcription of topoisomerase genes (18, 19, 29, 30). In the present study, we investigated the regulation of genes encoding two topoisomerases—topoisomerase I (TopA) and gyrase—and the role of their reciprocal dependence in maintaining topological homeostasis in S. coelicolor. We focused on the expression of topoisomerase genes in response to changes in the TopA level and to the inhibition of gyrase by use of novobiocin. Moreover, we analyzed the changes in topoisomerase level during adaptation to temperature stress. We demonstrate that transcriptional regulation of topoisomerases allows for the efficient maintenance of topological homeostasis in these industrially important bacteria. In addition, due to the topology-dependent gene expression, a better understanding of topoisomerase activity regulation in Streptomyces will contribute to the optimization of secondary metabolite production.

## MATERIALS AND METHODS

### Bacterial strains, plasmids, and growth conditions.

Basic DNA manipulation procedures were performed according to standard protocols (38). Unless otherwise stated, all enzymes and isolation kits were supplied by Thermo Scientific. Bacterial media and antibiotics were purchased from Difco Laboratories (Detroit, MI) and Carl Roth, respectively. The growth conditions and antibiotic concentrations, as well as conjugation to S. coelicolor, followed the general procedures described by Kieser et al. (39). For cultures treated with novobiocin, we used the antibiotic at concentrations (<20 μg/ml) that slowed the growth of S. coelicolor in liquid cultures but did not inhibit it completely. The S. coelicolor strains used in this study are shown in Table 1.

**TABLE 1:** S. coelicolor strains used in this study

Strain	Relevant genotype or description	Source or reference
M145	SCP1^−^ SCP2^−^	64
PS04	M145 Δ*topA*::*scar attB*ΦC31::pIJ6902*topA*	32
PS07	M145 *attB*ΦC31::pIJ6902*topA*	32
MSz-H1	M145 Δ*topA*::*xylE attB*ΦC31::pIJ6902*topA*	This study
MSz-1a	M145 Δ*topAp*_2_::Apra^r^	This study
MSz-4	M145 Δ*topAp*_1_	This study
MSz-5	PS07 Δ*topAp*_1_ *attB*ΦC31::pIJ6902*topA*	This study
MG01	M145 *attB*ΦBT1::pFLUXH	This study
MG02	M145 *attB*ΦBT1::pFLUXH*ermE*p	This study
MG03	M145 *attB*ΦBT1::pFLUXH*topA*p	This study
MG04	PS04 *attB*ΦBT1::pFLUXH*topA*p	This study
MS10	M145(pWHM3Hyg)	This study
MS11	PS04(pWHM3Hyg)	32
MS12	PS07(pWHM3Hyg)	This study

To inactivate the *topAp*_2_ promoter on the S. coelicolor chromosome, we replaced the region between bp −147 and −162 upstream of the *topA* gene with an apramycin resistance cassette (Apra^r^-*oriT*), yielding the MSz-1a (*topA*Δp2::Apra^r^) strain. First, the Apra^r^-*oriT* cassette was amplified from the pIJ773 vector by using the oligonucleotides prom2_PacI_FWD and prom2_PacI_RV and was subsequently introduced into the H5 cosmid by use of the Redirect system (40). The obtained cosmid was conjugated to strain M145, apramycin-resistant and kanamycin-sensitive exconjugants were selected, and the mutation was confirmed by PCR with oligonucleotides flanking the cassette insertion site. In the *topA*Δp2::Apra^r^ strain, transcription of the apramycin resistance gene is driven in the direction opposite that of the *topA* gene, which guarantees that transcription of the apramycin resistance gene does not affect *topA* transcription.

The *topAp*_1_ promoter was inactivated by disrupting the putative −10 region; 15 nucleotides in this region were replaced with the PacI restriction site sequence (TTAATTAA). To this end, we first constructed an H5 cosmid in which the −10 sequence was replaced with the Apra^r^-*oriT* cassette (amplified using the oligonucleotides prom1_PacI_FWD and prom1_PacI_RV) flanked by PacI restriction sites. Next, the H5 *topAp*_1_::Apra^r^-*oriT* cosmid was digested with the PacI restriction enzyme and religated to yield the H5 cosmid *topAp*_1_::PacI. To enable conjugative transfer of the H5 *topAp*_1_::PacI cosmid, the ampicillin resistance cassette was replaced with an Apra^r^-*oriT* cassette (32). The newly generated construct was conjugated into the *topA*Δp2::Apra^r^ strain. To facilitate double crossover, Kan^r^ exconjugants were restreaked on soya flour medium (SM) plates without antibiotics and subsequently screened for Kan^s^ and Apra^s^ colonies, which indicated the exchange of the *topAp*_2_::Apra^r^-*oriT* plasmid to yield the MSz-4 strain (*topA*Δp1::PacI). The strain was verified by sequencing of the *topA* promoter region. The MSz-5 strain was obtained by transformation of the *topA*Δp1::PacI strain with the pIJ6902*topA* plasmid (32).

To obtain S. coelicolor strains for analysis of the *topA* promoter (*topAp*) via reporter gene expression, we modified the previously described PS04 strain (containing a deletion at the native *topA* locus and with *topA* expressed in *trans* from the integrating vector pIJ6902) or the merodiploid strain PS07 (containing a second copy of *topA* delivered in *trans* in the integrating vector pIJ6902) (32). The strain MSz-H1 was constructed by modifying the PS07 strain (M145 pIJ6902*topA*), with the native copy of the *topA* gene being replaced by the *xylE-hyg-oriT* cassette. The *xylE-hyg-oriT* cassette consisted of two independently amplified DNA fragments: the *xylE* gene (amplified with the oligonucleotides H5*xylE*_FW and PacI*xylE*_RV from the pXE4 vector [41]) and an *hyg-oriT* fragment (amplified with the oligonucleotides PacIFRT_FW and *3543*RP1 from the pIJ10700 vector).

To quantify *topA* promoter activity, we examined luciferase reporter gene expression under the control of the *topA* promoter in strains MG04 and MG01, which were generated by introducing a pFLUX vector derivative into the wild-type (M145) and PS04 strains, respectively (42). The pFLUX vector was first modified by exchanging the apramycin resistance cassette for *hyg-oriT*, yielding the pFLUXH plasmid. We subsequently amplified a 450-bp region upstream of the *topA* gene, flanked with BamHI and NdeI restriction sites, and ligated this fragment into the pFLUXH vector digested with the same enzymes. Due to the use of the NdeI restriction site, we exchanged the native AAGTTG sequence with CATATG and replaced the native *topA* TTG translational start codon with an ATG start codon. We also constructed a version of the pFLUXH plasmid in which the 330-bp *ermE* promoter from Streptomyces erythraeus (35) was flanked by recognition sites for KpnI and NdeI and cloned into the vector. pFLUXH*topA*p, pFLUX*ermE*p, and the promoterless pFLUXH plasmid (used for data normalization) were conjugated to wild-type M145 and PS04 (Δ*topA* pIJ6902*topA*) (32) and were screened for Hyg^r^ exconjugants (Table 1).

### Reporter plasmid isolation.

The pWHM3Hyg plasmid (5.9 kbp) (32) was isolated using alkaline lysis and column purification based on a modified version of the manufacturer's (Syngen) procedure. After 48 h of growth in liquid 79 medium (43) supplemented with hygromycin, S. coelicolor hyphae were collected by centrifugation and suspended in the manufacturer's buffer containing 25 mg/ml of lysozyme. Hyphae were incubated at 30°C (or 42°C if isolated from heat-shocked cells) for 5 min. The subsequent steps followed the manufacturer's protocol. The isolated reporter plasmids were resolved in 0.8% agarose in Tris-acetate-EDTA (TAE) buffer in the presence of 4.6 μM chloroquine at low voltage. To visualize topoisomers, the gel was stained with ethidium bromide for 30 min at room temperature. The superhelical density (σ) was calculated as the change in linking number for relaxed and partially supercoiled plasmids (ΔLk) divided by the linking number for relaxed plasmids (Lk = molecule length [in base pairs]/10.4).

### Reporter gene activity assays.

To quantify the activity of catechol 2,3-dioxygenase (xylanase; the product of the *xylE* gene [41]) under the control of the *topA* promoter (41), crude extracts were prepared from 5-ml liquid cultures of the MSz-H1 strain (grown for 24 h in 79 medium). Changes in chromosomal supercoiling density were achieved by inducing expression of the *topA* gene with thiostrepton. Cell pellets were collected by centrifugation and washed twice with 20 mM KH_2_PO_4_ (pH 7.2) and then were resuspended in 500 μl of buffer A (100 mM KH_2_PO_4_, pH 7.5, 10% acetone, 20 mM EDTA) and sonicated. Cell extracts were supplemented with Triton X-100, incubated on ice for 15 min, and centrifuged. Xylanase activity was measured by the addition of 50 to 200 μl cell lysate to 1 ml 100 mM KH_2_PO_4_, pH 7.5, supplemented with 0.2 mM catechol at 30°C. Enzyme activity (in milliunits) was monitored by the increase in absorption at 375 nm and was subsequently normalized to the total protein used in the assay.

To measure luciferase activity, strains containing the *luxCDAEB* operon under the control of the *topA* or *ermE* promoter in the pFLUXH ΦBT1 integrating vector (MG01, MG02, MG03, and MG04) were used. The *luxAB* genes encode luciferase, whereas the *luxCDE* genes encode enzymes necessary for luciferase substrate (i.e., tetradecanal) biosynthesis. The strains MG01 and MG04 were cultivated in 79 medium with an appropriate concentration of thiostrepton (*topA* transcription inducer) for 24 h at 30°C. Subsequently, the cells were collected by centrifugation, weighed, and resuspended in 300 μl of 79 medium. Measurement of the luciferase activity, which was linearly correlated with *topA* promoter activity, was performed directly from cell pellets by use of an EnVision multilabel plate reader (PerkinElmer). The luminescence intensity was calculated for 100 mg of wet weight.

### RNA isolation and RT-qPCR.

RNA was isolated from S. coelicolor after 24 to 48 h of growth in liquid 79 medium not supplemented with glucose by use of a GeneJET RNA isolation kit according to the manufacturer's procedure. To increase the efficiency of RNA extraction, the cells were treated with a lysozyme solution (10 mg/ml) added to the suspension buffer prior to lysis. Isolated RNA was digested with DNase I (Thermo Scientific) to remove traces of chromosomal DNA and then was purified and concentrated using a GeneJET RNA cleanup and concentration microkit. Isolated RNA was tested for DNA contamination by PCR with oligonucleotides complementary to the S. coelicolor topA gene. The lack of a visible product after 30 PCR cycles with RNA samples as templates proved that they were DNA-free. One microgram of RNA was used for cDNA synthesis with an iScript reverse transcription (RT) kit (Bio-Rad). cDNA samples were subsequently diluted to a final concentration of 60 ng/μl and used for quantitative PCR (qPCR) (iTaq Universal SYBR green supermix; Bio-Rad). The level of a given transcript was quantified using *hrdB* as a reference gene (ΔΔ*C_T_* method) (see Fig. S7 in the supplemental material) and/or compared directly with a standard curve based on the purified H5 cosmid (StepOne Plus real-time PCR system; Applied Biosystems). For more details, see the MIQE form in the supplemental material. In the nested RT-PCR experiment, the RT3543RV oligonucleotide was combined with each of the forward oligonucleotides complementary to the *topA* upstream region (see Table S1). After 30 cycles of PCR, the products were resolved in a 1% agarose gel and stained with ethidium bromide.

### S1 nuclease mapping.

For S1 nuclease mapping, RNA samples were isolated from solid SM cultures grown for 18 to 96 h at 30°C according to a protocol described by Aínsa et al. (44). S1 nuclease protection assays were performed as described by Kelemen et al. (26), using 30 μg of RNA. The oligonucleotides 3543_400Fw and 3543_77RV were used to amplify the *topA* promoter region from chromosomal DNA isolated from the M145 strain. The amplified region was subsequently cloned into the pGEM T-Easy vector (Promega) and used as a template for a PCR using M13FW and the radioactively labeled oligonucleotide 3543_77RV. The isolated RNA was hybridized with a DNA probe radioactively labeled at the 5′ end by use of phage T4 polynucleotide kinase and [γ-^32^P]ATP. Control S1 nuclease digestions were carried out with an *hrdB* probe, which was used as a combined semiquantitative standard and a control of RNA quality. After purification, the RNA-probe complex was digested with S1 nuclease.

### SDS-PAGE and Western blotting.

Cell lysates were separated in 10% SDS-polyacrylamide gels according to standard procedures. After electrophoresis, proteins were transferred to a nitrocellulose membrane and blocked with 2% milk in Tris-buffered saline plus Tween 20 (TBST). The blots were subsequently incubated with polyclonal TopA antiserum (1:10,000) and visualized with alkaline phosphatase-conjugated goat anti-rabbit antibodies (1:5,000) (32). The band intensities were analyzed using ImageJ2x software. To determine the optimal amount of crude cell lysate, we quantified the band intensities and selected the range wherein band intensity correlated linearly with protein concentration (see Fig. S9 in the supplemental material).

## RESULTS

### Two promoters control transcription of the *topA* gene in S. coelicolor.

**FIG 1:**
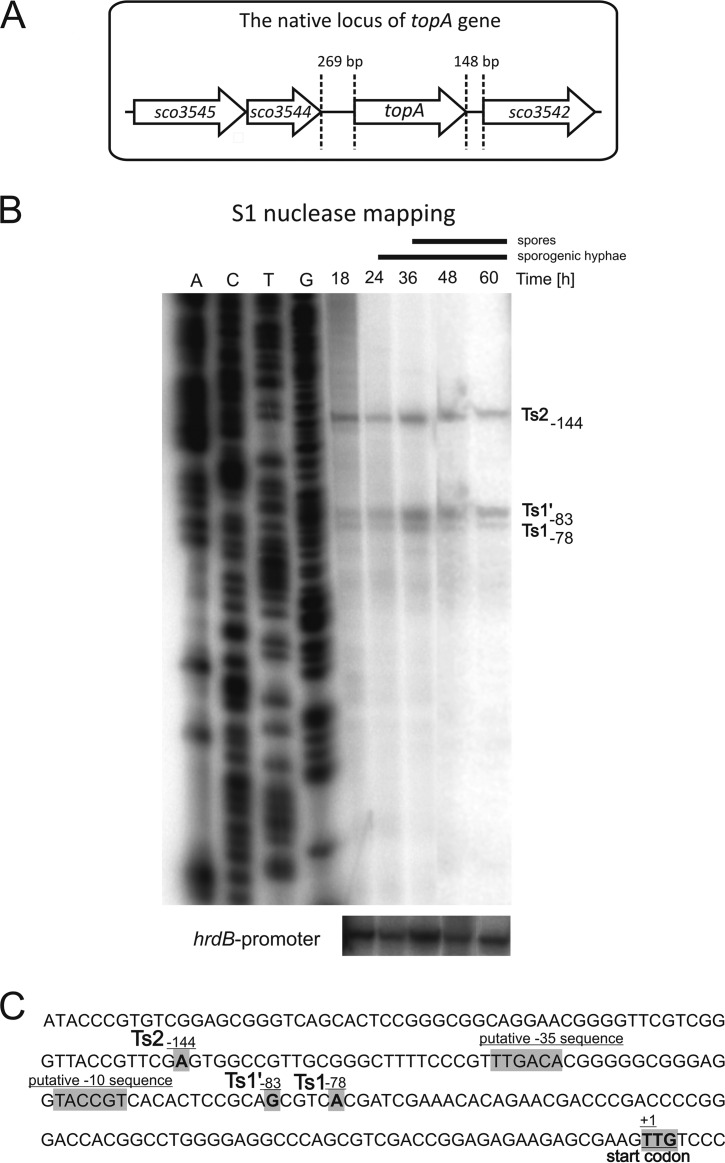
Identification of *topA* transcriptional start sites during S. coelicolor differentiation. (A) Map of the S. coelicolor chromosomal region in the vicinity of the *topA* gene (*sco3543*). (B) S1 nuclease mapping of *topA* transcripts. S1 mapping of the *hrdB* promoter region served as the mRNA control. (C) Sequence of the region upstream of *topA*. The identified Ts (transcription start) sites, the TTG translational start codon, and putative *topAp*_1_ promoter elements are marked in gray.

Streptomyces species, as nonmotile soil bacteria, are likely to be exposed to environmental stresses that induce sporulation and antibiotic production, both of which are associated with changes in chromosomal topology. Moreover, in contrast to most bacteria, except for several actinobacterial species, Streptomyces species possess only one type I topoisomerase, TopA. Thus, we expected that the level of TopA should be tightly regulated. Because the first major step in the regulation of gene expression is the control of promoter activity, we set out to investigate the region upstream of the *topA* gene. The gene encoding TopA (*sco3543*) is located in a central, evolutionarily conserved region of the S. coelicolor linear chromosome. Interestingly, translation of the *topA* gene begins at a rare TTG codon, which is used as a start codon for only 3% of genes in S. coelicolor (45). The start codon is preceded by a relatively long (269 bp) intergenic region, suggesting that *topA* expression is independently regulated by a *topA*-dedicated promoter (Fig. 1A). To identify a transcriptional start (Ts) point(s) positioned upstream of the *topA* gene, S1 nuclease mapping was performed using RNAs from sporulating cultures (18 to 60 h of growth on SM medium). S1 mapping revealed three bands, suggesting the presence of 3 Ts sites. Two of these Ts sites—Ts1 and Ts1′—are positioned at bp −78 and −83 upstream of the TTG start codon, respectively, whereas an additional site, the Ts2 site, is located at bp −144 (Fig. 1B). The close proximity of the Ts1 and Ts1′ sites may suggest the existence of two overlapping promoters; however, the possibility of a single promoter with two transcription start points cannot be excluded at the moment. For the sake of simplicity, we refer to the regions upstream of the Ts1/Ts1′ and Ts2 sites as the p1 and p2 promoters, respectively. During the differentiation time course, all three bands were clearly visible, and their intensities were similar (Fig. 1B). The lack of a visible change in transcript levels at the time of formation of sporogenic hyphae and spores suggests the nearly constitutive expression of *topA*, with equal involvement of the p1 and p2 promoters, during S. coelicolor sporulation.

**FIG 2:**
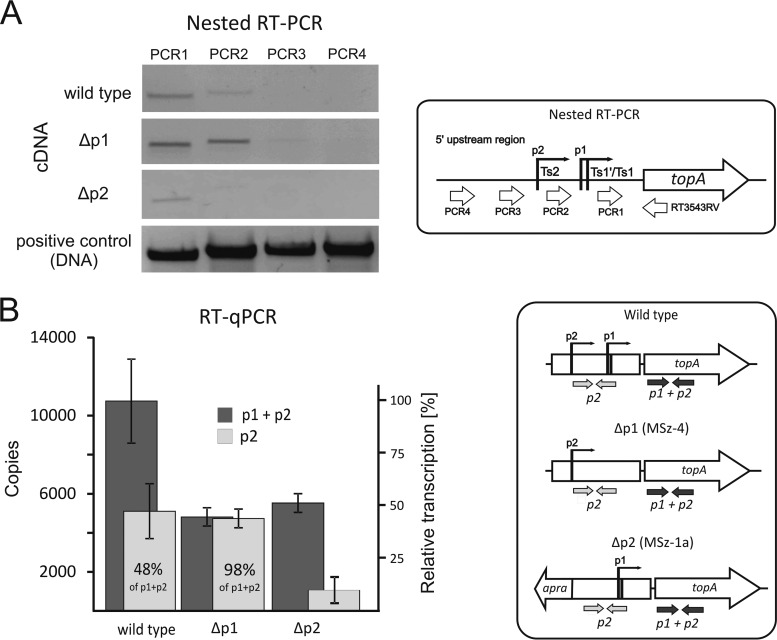
Contribution of the *topA* promoter to the total transcript level. (A) Nested RT-PCR using oligonucleotides complementary to the upstream region of the *topA* promoter (see the scheme at right). For the wild-type strain, the two bands (PCR1 and PCR2) correspond to the products of the p1 and p2 promoters (see the scheme at right). (B) RT-qPCR analysis of RNAs isolated from the wild-type strain and the p1 and p2 mutants. Oligonucleotides were designed to detect the overall *topA* transcript (p1 + p2) and p2-specific transcript (p2) levels (see the scheme at right).

To confirm the transcription of the *topA* gene from the two predicted promoters and to exclude the contribution of an additional upstream promoter(s), we examined transcript levels in the wild-type strain and in strains harboring mutations in one promoter during vegetative growth (S. coelicolor does not sporulate in liquid cultures). The nested RT-PCRs utilized different forward oligonucleotides and identified only transcripts downstream of the Ts1/Ts1′ (p1 transcript) and Ts2 (p2 transcript) sites (Fig. 2A). Additionally, we disrupted the p1 (MSz-4 strain; Δp1) and p2 (MSz-1a; Δp2) promoters (see Fig. S1A in the supplemental material). For the Δp1 strain, equal amounts of the PCR1 and PCR2 products were observed, confirming that all *topA* transcription in this mutant was driven by the p2 promoter (Fig. 2A). On the other hand, for the Δp2 strain, only the p1 transcript was detectable (Fig. 2A). Notably, we could not disrupt both promoters at the same time (see Fig. S1B), which, taking into account the indispensability of the *topA* gene (32), confirms that the promoters were inactivated by the introduced mutations. Our results suggest the absence of additional upstream transcriptional sites and readthrough transcripts derived from the *sco3544* gene under the conditions tested.

Next, we decided to further analyze the contributions of both promoters to the transcript levels and to dissect their regulation. Using RT-qPCR, we calculated the overall copy number of the *topA* transcript (p1 + p2) as well as the p2 promoter-specific transcript (p2). This analysis confirmed that the *topA* promoters were equally active (48% of the total transcripts were derived from p2, whereas 52% were from p1) during vegetative growth. In the Δp1 strain, the level of the *topA* transcript was only half the wild-type level (Fig. 2B), suggesting that p2 activity was not increased by p1 disruption. A similar situation was observed for the Δp2 strain, in which the level of *topA* transcription decreased to approximately 50% of the wild-type level following p2 promoter inactivation. Surprisingly, although the introduced modifications decreased the *topA* transcript level, we did not observe any inhibition of the growth rate or differentiation of the mutant strains under standard conditions (see Fig. S2 in the supplemental material).

These results show that during S. coelicolor differentiation, the *topA* gene is transcribed at a nearly constant level. Transcription of the *topA* gene is controlled by two equally active promoters: p1 and p2. Interestingly, the elimination of one, but not both, of the promoters is possible without any visible effect on growth or change of the activity of the other, intact promoter.

### The p1 promoter is highly induced by increased supercoiling.

**FIG 3:**
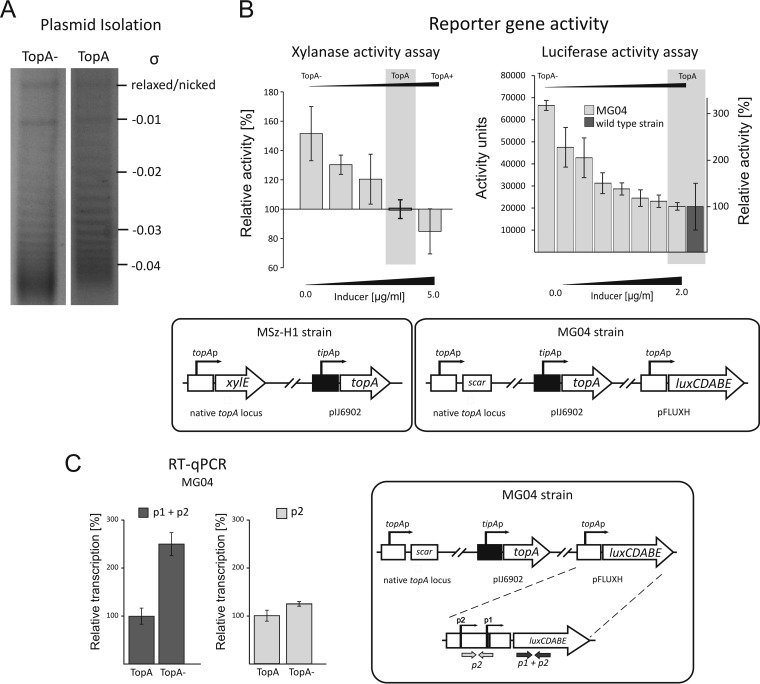
Induction of the *topA* promoter by high chromosomal supercoiling density. (A) Analysis of the supercoiling density (σ) of the reporter plasmid pWHM3Hyg isolated from strains with a wild-type (TopA) or depleted (TopA−) level of topoisomerase I. (B) (Left) Relative xylanase activities in MSz-H1 strain cell extracts (see the scheme at bottom) cultured under different inducing conditions, i.e., high, wild-type, and low DNA supercoiling densities. Different supercoiling conditions were achieved via the induction of *tipAp*-controlled *topA* expression with 0 to 5 μg/ml of thiostrepton, as indicated. The wild-type level of TopA protein corresponds to an inducer concentration of 2 μg/ml, whereas lower and higher inducer concentrations led to TopA depletion (TopA−) and overproduction (TopA+), respectively. (Right) Relative luciferase activities in the MG04 strain (see the scheme at bottom) cultured under conditions inducing high supercoiling density. High supercoiling in MG04 was achieved by the induction of *tipAp*-controlled *topA* gene expression with thiostrepton at concentrations of up to 2 μg/ml (wild-type level of TopA protein) and was compared to that of the wild-type strain (MG03). (C) Levels of *topA* promoter-driven transcripts in the MG04 strain (see scheme at right) with TopA depletion (TopA−) and wild-type TopA levels (TopA). The overall *topA* promoter activity (p1 + p2) was quantified by detection of the *luxC* transcript (left), whereas p2-specific transcription (p2) was quantified using oligonucleotides specific to the *topA* promoter region (right).

Studies on E. coli, M. smegmatis, Streptococcus pneumoniae, and Haemophilus influenzae showed that, in bacteria, changes in chromosomal supercoiling density affect the expression of many genes, including *topA* (11, 19, 46–48). Thus, we expected that changes in DNA supercoiling induced by alteration of the TopA level in S. coelicolor might also affect its own gene transcription. To analyze the sensitivity of the *topA* promoter to DNA supercoiling, we used the previously constructed PS04 strain, which has an inducible TopA level (the *topA* gene is under the control of the inducible *tipA* promoter [32]). The depletion of TopA resulted in increased negative supercoiling of the reporter pWHM3Hyg plasmid (Fig. 3A; see Table S2 in the supplemental material). The PS04 strain was subsequently modified by the introduction of reporter genes—*xylE* at the native *topA* locus (encoding xylanase in the MSz-H1 strain) and *luxCDABE* at the phage attachment site (encoding luciferase in the MG04 strain)—under the control of the *topA* promoter. The obtained strains allowed us to quantify the transcriptional activity of the *topA* promoter under increased supercoiling conditions and to verify the influence of the chromosomal locus on promoter activity.

TopA depletion (by the absence of the inducer) (Table 1) highly induced the *topA* promoter. The activity of the *xylE* reporter gene product increased up to 1.5-fold compared to its activity in the control culture (i.e., the native TopA level). The increase of *topA* promoter activity in the MSz-H1 strain was proportional to the decrease of the cellular level of TopA (Fig. 3B, left panel). The strong induction of the *topA* promoter with a lowered intracellular TopA level was confirmed by direct measurement of luciferase activity. Moreover, this strain allowed us to further investigate the activity of the *topA* promoter positioned outside the central core region of the linear chromosome. At the lowest cellular TopA level (in the absence of the inducer), the activity of the *topA* promoter was 3-fold higher than that under standard conditions (Fig. 3B, right panel). An increase in TopA level (with an inducer concentration of 0.05 to 2.0 μg/ml) resulted in a decrease in luciferase activity. At the wild-type TopA level, we observed luciferase activity in the MG04 strain (with an inducer concentration of 2 μg/ml) that was comparable to that in the reference strain (MG01; wild type). A further increase of the inducer concentration did not affect the luciferase activity (not shown).

Next, we asked whether one or both *topA* promoters were sensitive to increased DNA supercoiling. To this end, we used the MG04 strain, in which the *topA* promoter drives transcription of the *luxCDABE* operon, to quantify the transcription of the first gene in the operon (*luxC*). By comparing transcription levels driven by the *topA* promoter at the low topoisomerase I level (TopA^−^; MG04) and the wild-type level (TopA; MG04 plus 2.0 μg/ml of the inducer), we confirmed a >2-fold increase of the overall level of the p1 + p2 transcript (Fig. 3C) in the TopA depletion background. Interestingly, we determined using p2-specific oligonucleotides that the depletion of topoisomerase I did not induce transcript expression from the *topAp*_2_ promoter (Fig. 3C).

**FIG 4:**
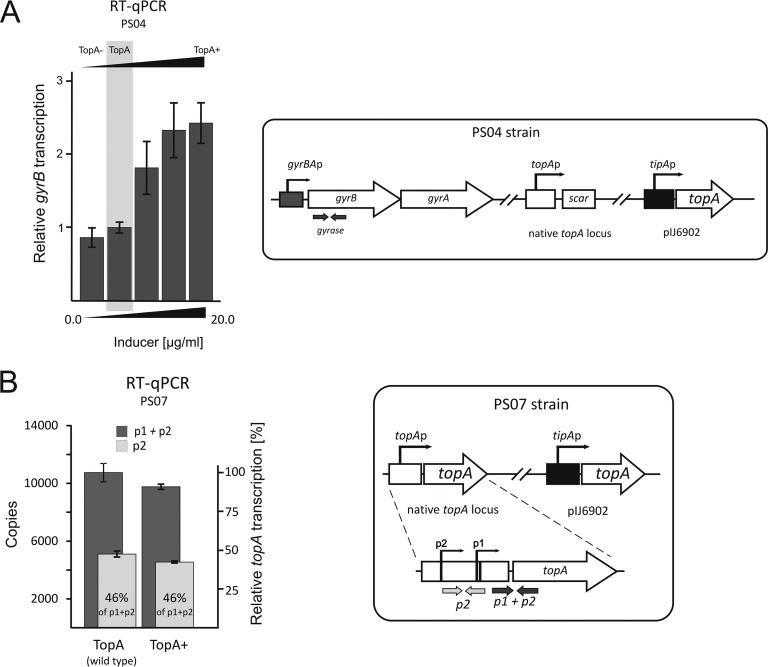
Influence of increased TopA level on topoisomerase gene transcription. (A) RT-qPCR analysis of the *gyrB* transcript level in the PS04 strain with high (TopA+), wild-type (TopA), and depleted (TopA−) levels of topoisomerase I. (B) *topA* transcript levels in a TopA-overproducing strain (PS07; see the scheme at right). Oligonucleotides were designed to detect the p2 transcript (p2) and the overall *topA* transcript (p1 + p2) specific to the *topA* promoter (*topAp*), while discriminating the transcripts from that from the *tipA* promoter (*tipAp*).

It has been demonstrated that supercoiling conditions affect both *topA* and gyrase gene transcription in a number of bacterial species (11, 30, 46, 49), thereby providing a mechanism for maintaining topological balance. We expected that an elevated TopA level should be correlated with decreased gyrase gene transcription. An earlier transcriptional analysis indicated that the *gyrBA* operon in Streptomyces venezuelae and S. coelicolor is transcribed from a single promoter (50; Mark Buttner, personal communication). Interestingly, TopA depletion (TopA^−^) only slightly inhibited *gyrB* transcription (87% of wild-type activity) (Fig. 4A). This presumably explains the high chromosomal supercoiling density and growth inhibition of the TopA-depleted strain.

In summary, TopA depletion leads to an increase of negative DNA supercoiling and to strong induction of the *topA* promoter, but the p1 promoter is solely responsible for the induction of *topA* expression under high supercoiling conditions. Remarkably, gyrase gene transcription is only slightly inhibited by high supercoiling, and thus a lower gyrase level does not provide the mechanism that restores normal supercoiling.

### Increased transcription of the *gyrBA* operon compensates for TopA overproduction.

Interestingly, we noticed that in contrast to TopA depletion, its overproduction only slightly inhibited *topA* promoter activity. In the Msz-H1 strain, XylE activity with an elevated TopA level (with an inducer concentration of 5 μg/ml; TopA^+^) was reduced only to approximately 85% of the level observed in the control culture (TopA; native enzyme level) (Fig. 3B, left panel). This observation was confirmed using the PS07 strain cultured at a high *tipAp* inducer concentration (5.0 μg/ml) (see Fig. S3 in the supplemental material). As observed previously, increasing the TopA level only slightly decreased *topA* expression, and 82% of promoter activity was retained in the control noninduced strain (TopA; native level) (Fig. 4B). Surprisingly, the ratio of p1 to p2 transcripts remained constant under conditions with an elevated TopA level; the p2 transcript constituted 46% of the total transcripts generated both before and after induction (Fig. 4B). Thus, the increased TopA level only slightly reduced the activities of both the p1 and p2 promoters. The lack of efficient inhibition of the *topA* gene by TopA overproduction may be surprising, but the analysis of reporter plasmid topoisomer distribution in the gel confirmed that an increased level of TopA only slightly affected overall DNA supercoiling (see Fig. S4 and Table S2).

The observation that TopA depletion led to increased DNA supercoiling and activation of the *topA* promoter while TopA overproduction did not result in significant inhibition of the *topA* promoter suggests that an increase of the TopA level may be compensated by changes in the level of gyrase. We therefore investigated whether the transcription of the *gyrBA* operon was upregulated by TopA overproduction (TopA^+^; inducer concentration of >2 μg/ml in strain PS04). Indeed, the increased level of TopA stimulated the *gyrBA* promoter 2- to 3-fold compared to its activity with the wild-type TopA level (Fig. 4A).

Thus, we observed a significant increase of gyrase gene expression induced by TopA overexpression and only limited downregulation of the *topA* promoter. We concluded that since TopA overproduction is not accompanied by silencing of the *topA* promoter, the major mechanism that prevents chromosomal relaxation is based on upregulation of the *gyrBA* operon and an increase of the gyrase level.

### Activation of the *gyrBA* operon restores chromosomal supercoiling after novobiocin treatment.

Changes in *topA* and *gyrBA* promoter activity in strains with a modified TopA level suggested that the transcription of both topoisomerase genes is supercoiling sensitive. To exclude the possibility that TopA regulates its own gene and/or gyrase gene expression via direct binding of the promoter regions, we used novobiocin, an inhibitor of bacterial gyrase. Novobiocin blocks the binding of ATP by the GyrB subunit and inhibits the introduction of negative supercoils by the enzyme (51, 52), thereby promoting chromosomal relaxation.

**FIG 5:**
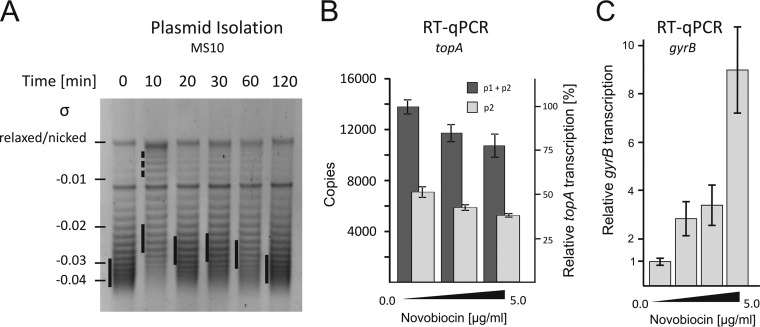
Changes in chromosomal supercoiling and *topA* and *gyrB* gene transcription after novobiocin treatment. (A) Analysis of the supercoiling density (σ) of the reporter pWHM3Hyg plasmid isolated from the wild-type strain cultured for up to 120 min in the presence of novobiocin (5 μg/ml). (B) Relative transcription levels of p2-specific (p2) and overall (p1 + p2) *topA* transcripts in the wild-type strain in the presence of novobiocin (up to 5.0 μg/ml). (C) RT-qPCR analysis of *gyrB* transcript levels in the wild-type strain in the presence of increasing concentrations of novobiocin (up to 5.0 μg/ml).

First, we investigated how exposure to novobiocin influences the supercoiling of the reporter pWHM3Hyg plasmid. Immediately (10 min) after addition of novobiocin (5 μg/ml), the negative supercoiling density (σ) of the plasmid decreased significantly, suggesting DNA relaxation. Subsequently, the plasmid supercoiling increased, reaching the wild-type level up to 120 min after the exposure to novobiocin (Fig. 5A; see Table S2 in the supplemental material). This observation suggests that the rapid DNA relaxation due to gyrase inhibition activates the mechanism that restores the appropriate chromosomal supercoiling density, presumably involving changes in topoisomerase levels. To identify the changes of topoisomerase gene expression which maintain topological balance upon gyrase inhibition, we analyzed the transcription of the *topA* gene as well as the *gyrBA* operon in strains cultured in the presence of novobiocin.

Inhibiting gyrase led to a slight inhibition of *topA* transcription. At the highest tested novobiocin concentration (5 μg/ml), we detected decreases in both p1 + p2 and p2 transcripts, to 78% and 83% of their initial levels, respectively (Fig. 5B). The ratio between the transcripts was retained, suggesting that both the p1 and p2 promoters were partially inhibited in the presence of novobiocin. We also observed that the *topA* promoter-driven reporter gene transcription in the wild-type background (strain MG03) was only slightly or not sensitive to novobiocin (see Fig. S5A in the supplemental material), confirming our previous observation that TopA overproduction does not inhibit the *topA* promoter.

In contrast to that from the *topA* promoter, the transcription of the *gyrBA* operon was elevated 9-fold (Fig. 5C) in the presence of novobiocin (1 to 5 μg/ml of novobiocin). The high activation of the gyrase genes confirms the supercoiling sensitivity of their promoter and explains the return to close-to-normal supercoiling shortly after novobiocin-induced chromosomal relaxation.

In summary, we found that *topA* downregulation is only marginally involved in the return to normal chromosomal supercoiling upon novobiocin treatment, similar to the results observed earlier for TopA induction. We showed that gyrase overproduction is the predominant mechanism for restoring the supercoiling level after induced chromosomal relaxation.

### Heat shock adaptation involves changes in topoisomerase I and gyrase levels.

After exposure to stress factors (e.g., heat shock), the transcription of a number of genes changes to facilitate cellular adaptation and survival under new environmental conditions. It has been shown previously that changes in gene transcription profiles are mediated by alterations in chromosomal supercoiling (11, 13).

**FIG 6:**
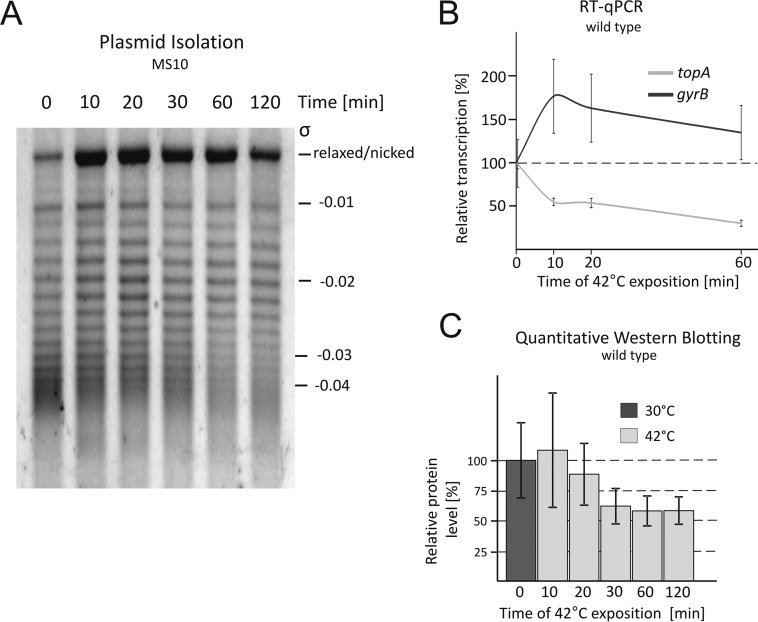
Changes in chromosomal supercoiling and *topA* and *gyrB* gene expression under temperature stress conditions. (A) Analysis of the supercoiling density (σ) of the reporter pWHM3Hyg plasmid isolated from the wild-type strain exposed to 42°C for up to 120 min. (B) Relative transcription levels of the *topA* and *gyrB* genes after exposure to 42°C. The level of each gene in the control strain (i.e., wild type) was set at 100%; any changes in transcription were compared to the wild-type level. (C) Quantitative Western blot determination of the TopA level during incubation at 42°C.

To answer the question of how S. coelicolor chromosomal supercoiling is affected by heat shock, we analyzed the changes in supercoiling of the reporter plasmid after exposure to 42°C. Immediately (10 min) after temperature upshift, the DNA negative supercoiling density (σ) decreased significantly, and the relaxed form was observed to be the most dominant form. The longer incubation at an elevated temperature resulted in a gradual increase of plasmid supercoiling (Fig. 6A).

To determine the changes in topoisomerase levels accompanying the adaptation to heat shock, we characterized the expression of genes encoding TopA and gyrase. Since gyrase expression was also sensitive to supercoiling, we expected that the temperature-induced chromosomal relaxation should affect *gyrBA* promoter activity. Indeed, the RT-qPCR analysis showed that upon exposure of cells to 42°C, the *gyrB* transcript level first increased notably, to 175% of the native level after 10 min of heat shock, and then decreased slowly and stabilized at 150% of the native level under standard growth conditions (Fig. 6B). Next, we established that shortly (10 min) after exposure to 42°C, the *topA* transcript level dropped to 50% of the initial level (Fig. 6B), resulting in downregulation of both the p1 and p2 promoters (see Fig. S6 in the supplemental material). The efficient downregulation of *topA* transcription was confirmed by measuring *luxCDABE* reporter gene expression (see Fig. S5B). Subsequently, we determined the protein level by quantitative Western blotting (Fig. 6C). The decrease in TopA level was observed after only 20 min of exposure to 42°C, and after 60 min, the protein level had reached nearly half of the initial level. Thus, at an elevated temperature, the decrease in *topA* transcription directly precedes a decrease in protein expression, suggesting that at the cellular level, TopA is regulated at the transcriptional stage rather than by posttranslational processing.

In summary, we demonstrated that high temperature induces rapid chromosomal relaxation. Shortly after heat shock, the topoisomerase I and gyrase levels were significantly decreased and increased, respectively. The coordinated action of both enzymes presumably results in adaptation to stressful conditions.

## DISCUSSION

As a response to environmental factors and the cell cycle, DNA supercoiling serves as a global gene transcriptional regulator in bacteria (11, 46). The maintenance of topological homeostasis requires coordination of the opposing activities of the enzymes gyrase and topoisomerase I (TopA), which act by the introduction and removal of negative supercoils, respectively. Thus, changes in the TopA and/or gyrase level as well as ATP availability for gyrase modulate the supercoiling state of the bacterial chromosome. Here we describe the interplay between TopA and gyrase activities in the multigenomic bacterium Streptomyces coelicolor. In response to environmental stressors, such as nutrient limitation, S. coelicolor sporulates and produces secondary metabolites. It has been shown that changes in the TopA level affect sporulation as well as secondary metabolism (32).

In S. coelicolor, *topA* transcription is driven by at least two promoters that are constitutively active throughout development. S1 nuclease mapping of the *topA* promoter confirmed previous RNA microarray data obtained by Nieselt et al. (53), and our analysis of earlier 5′-end mapping data (differential transcript analysis) (50) identified the Ts1 site as a major *topA* gene transcriptional start site. The Ts1′ and Ts2 sites were also detected, but due to their low signals they were regarded as statistically irrelevant (see Fig. S7 in the supplemental material). However, our S1 nuclease mapping clearly showed the presence of both transcriptional start points. A comparison of the *topA* upstream region and other described S. coelicolor promoters suggested the presence of −35 and −10 sequences in the p1 promoter similar to sequences recognized by the HrdB protein, a homologue of the E. coli sigma^70^ factor (54). The involvement of the constitutively produced HrdB sigma factor in the expression of the p1 promoter corroborates the constant level of *topA* transcription observed during the cell cycle. Interestingly, the spacer between the identified −35 and −10 regions in the p1 promoter is extremely rich in GC pairs and composed of 13 nucleotides rather than 17 or 18 bp, which is the optimal length for the binding of sigma factors (54, 55). Thus, in S. coelicolor, the *topAp*_1_ promoter may resemble supercoiling-sensitive promoters described for other species, e.g., those for *flaA* in Helicobacter pylori (30) and *topA* in M. smegmatis and M. tuberculosis (19), as well as stress response *proU* and *recA* promoters in E. coli (56) and the promoter of pathogenicity *ssrAB* genes in Salmonella enterica (57).

The activity of the p1 promoter (but not p2) increased notably when the TopA level was depleted. Thus, the regulation of the *topA* promoter is tuned to prevent excessive negative supercoiling by increasing the TopA level. Surprisingly, excessive supercoiling only slightly affected gyrase gene expression. This result is in contradiction with the negative-feedback regulation of the gyrase level in most bacterial species. The phenomenon of relaxation-stimulated transcription of gyrase genes has also been described previously (11, 29, 46). Moreover, it was recently shown that in the Chlamydiae, gyrase genes can be regulated by positive stimulation in response to increasing negative supercoiling (49). Analysis of topoisomerase gene expression suggested that in S. coelicolor, the main mechanism preventing excessive negative supercoiling employs the induction of TopA and is only slightly dependent on gyrase gene downregulation.

In contrast to the limited responsiveness to increased supercoiling, the promoter of the *gyrBA* operon in S. coelicolor is highly induced by increased chromosomal relaxation, such as that induced by TopA overproduction or novobiocin treatment. However, the increased relaxation has a weak negative influence on the S. coelicolor topA gene, i.e., the overproduction of TopA slightly inhibits both p1 and p2 activities. Such regulation is remarkably different from the efficient negative-feedback mechanism of *topA* expression that has been described for other bacterial species (18, 19, 46, 49). In the closely related species M. smegmatis and M. tuberculosis, *topA* transcription (also controlled by two promoters) is very efficiently inhibited by chromosomal relaxation induced by novobiocin treatment or TopA overproduction (19). The differences in the regulation of the *topA* promoters in Mycobacterium and Streptomyces might be explained by the contribution of the transcription of neighboring genes. For Mycobacterium, it was shown that the transcription of the upstream gene overlaps that of the *topA* gene (19). For S. coelicolor *topA* gene transcription, we excluded the contribution of upstream promoters. It seems plausible that the differences in *topA* regulation in M. smegmatis and S. coelicolor are connected to their chromosomal organization. Whereas the Mycobacterium chromosome is organized as a single copy of a circular chromosome that is segregated, as in other rod-shaped bacteria, during its replication, Streptomyces hyphal cells contain multiple copies of linear chromosomes. In S. coelicolor, vegetative hyphal chromosomes are decondensed, and their replication is not followed by active separation. It seems possible that maintaining increased relaxation of tens of chromosomes, which presumably requires TopA activity, prevents them from tangling and enables their distribution in the elongated hyphal cells (32). Thus, the high responsiveness of the *topA* promoter to an excess of negative supercoiling may be part of a strategy that favors chromosomal decondensation. It also cannot be excluded that weak negative-feedback regulation of the transcriptional level is complemented by posttranslational downregulation of the TopA level in S. coelicolor to prevent excessive relaxation (58), whereas upregulation of the gyrase level apparently provides a main mechanism that prevents excessive chromosomal relaxation.

We showed that exposure to thermal stress induces rapid reporter plasmid relaxation in S. coelicolor, followed by a gradual increase of negative supercoiling. The transitory relaxation of the reporter plasmid upon thermal stress is well described for E. coli (59, 60). Soini et al. (61) showed for E. coli that immediately after a temperature upshift, both ATP and ADP levels increased rapidly and transiently changed the ATP/ADP ratio, which thus may influence gyrase activity. The immediate relaxation may also be explained by the change in gyrase activity, which can relax DNA in an ATP-independent manner under heat shock conditions. It seems possible that a similar mechanism of transient DNA relaxation may contribute to adaptation to stress conditions in S. coelicolor. Additionally, the unusually high processivity of S. coelicolor TopA (37) presumably accounts for rapid relaxation of the chromosome under conditions of lower gyrase activity.

The fraction of relaxed plasmid observed shortly after S. coelicolor heat shock gradually decreased, and this was associated with upregulation of gyrase transcription and downregulation of the *topA* gene. Since the *gyrBA* promoter is sensitive to low supercoiling density, as shown by novobiocin treatment, its upregulation by heat shock may be the result of transient chromosomal relaxation. Interestingly, the heat shock-induced downregulation of the *topA* promoter was much stronger than that resulting from TopA overproduction or novobiocin treatment, suggesting a relaxation-independent mechanism of promoter silencing. This possibly involves binding of an unknown repressor (however, binding of TopA to its own promoter cannot yet be excluded) that blocks *topA* transcription. Another possibility is based on the observation that the transcription of the *hrdB* gene decreases under thermal stress conditions (62) (see Fig. S8 in the supplemental material). However, DNA relaxation under heat shock conditions is extremely rapid and cannot be explained only by the decrease of the HrdB level. Thus, we speculate that in the first step, the HrdB affinity for the relaxed *topA* promoter may be lowered. In the next step, when *gyrBA* transcription is elevated, restoring topological homeostasis, the lowered HrdB level keeps the transcription of *topA* at a decreased level. Thus, the coordinated action of the *topA* and *gyrBA* promoters leads to gradual restoration of normal chromosomal supercoiling after its rapid relaxation. It is also possible that the low level of TopA during thermal stress is provided by proteolytic degradation. It has been suggested that in S. coelicolor, the TopA protein is potentially degraded by the Pup-proteasome system (58). This is a modification that is unique to actinobacteria and is based on attachment of a small ubiquitin-like Pup protein that is associated with proteasome-mediated protein degradation.

It has been shown that small changes of the topological balance are involved in adaptation to stress conditions. In E. coli, *topA* gene expression is precisely regulated by four distinct promoters, including a constitutive promoter as well as a heat shock-responsive element (18, 21). Moreover, an E. coli strain lacking the *topA* gene has been shown to be more sensitive to oxidative stress (24, 63). In pathogenic strains of H. pylori and Salmonella, discrete changes in supercoiling regulate the transcription of genes involved in host persistence (25, 30). The changes in chromosomal supercoiling after exposure to elevated temperatures reinforce the involvement of chromosomal topology in response to stress conditions, and this phenomenon is likely to contribute to heat shock adaptation in S. coelicolor.

**FIG 7:**
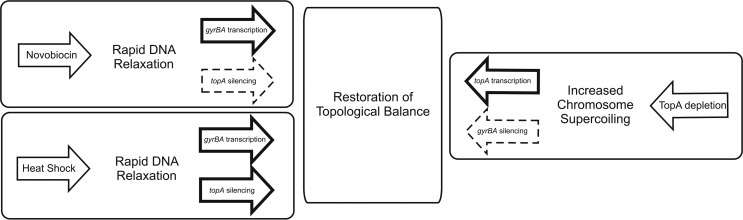
Topological homeostasis. The figure shows the restoration of topological balance after chromosomal supercoiling alteration due to changes in topoisomerase activities or exposure to elevated temperature. Bold arrows mark the dominant mechanism, whereas dashed arrows correspond to supporting pathways.

We have demonstrated here that in Streptomyces coelicolor, the promoters of the TopA- and gyrase-encoding genes are specialized to respond to different supercoiling states (Fig. 7). The *topA* gene is stimulated by high supercoiling density and is weakly sensitive to DNA relaxation, whereas gyrase expression is induced only under chromosomal relaxation conditions and is only slightly inhibited by increased supercoiling. Thus, in S. coelicolor, both the *topA* and *gyrB* genes are subject to positive regulation by a shift of chromosomal supercoiling. The negative-feedback regulation is relatively weak and may only precisely tune transcription rather than preventing the loss of topological balance (Fig. 7). However, the transcriptional downregulation of *topA* coupled to the upregulation of the gyrase genes is involved in the dramatic chromosomal compaction observed after thermal stress. Thus, we suggest that Streptomyces evolved a specific system of topological homeostasis maintenance that involves positive regulation of topoisomerase genes and presumably is connected with the stress response and with changes in chromosomal condensation during development. Insights into the Streptomyces topological homeostasis maintenance mechanism should prove useful for understanding topologically dependent gene expression, particularly in antibiotic pathways.

## Supplementary Material

Supplemental material
