# Supplementary material for: The Coordinated Positive Regulation of Topoisomerase Genes Maintains Topological Homeostasis in Streptomyces coelicolor
Source: J Bacteriol. 2016 Oct 7;198(21):3016–28. doi: 10.1128/JB.00530-16 (PMC5055605; doi:10.1128/JB.00530-16)
Supplement: Supplemental material [file supp_198_21_3016__index.html]

Supplemental material 

# The Coordinated Positive Regulation of Topoisomerase Genes Maintains Topological Homeostasis in Streptomyces coelicolor

## Supplemental material

- Supplemental file 1 -

  Table S1, oligonucleotides

  Table S2, characterization of *S. coelicolor* strains under different conditions

  Fig. S1, construction of mutants

  Fig. S2, growth and differentiation of mutants

  Fig. S3, analysis of TopA level

  Fig. S4, isolation of the reporter plasmid pWHM3Hyg

  Fig. S5, influence of novobiocin and heat shock on reporter gene expression

  Fig. S6, specific activities of *topA* promoters under heat shock conditions

  Fig. S7, identification of *topA* transcription start point

  Fig. S8, RT-qPCR analysis of *hrdB* gene transcription

  Fig. S9, quantitative western blotting

  RT-PCR data

  PDF, 1.3M
