# Supplementary material for: The Coordinated Positive Regulation of Topoisomerase Genes Maintains Topological Homeostasis in Streptomyces coelicolor
Source: J Bacteriol. 2016 Oct 7;198(21):3016–28. doi: 10.1128/JB.00530-16 (PMC5055605; doi:10.1128/JB.00530-16)
Supplement: Supplemental material [file JB.00530-16_zjb999094220so1.pdf]

Table S1

Table S1. Oligonucleotides

Oligonucleotide	Sequence	Description
H5*xylE*_FW	GGGGAGGCCCAGCGTCGACCGGAGAGAAGAGCGAAGTTGATGAACAAAGGTGTAATGCGAC	Construction of H1 strain
PacI*xylE*_RV	GGTTAATTAATCAGGTCAGCACGGTCATGA	Construction of H1 strain
PacIFRT_FW	GGTTAATTAAATTCCGGGGATCCGTCGACC	Construction of H1 strain
*3543*RP1	GTGGAGACGTGCGGAGGAGCGGGCGGGTCTGAGCCGGGCTGTAGGCTGGAGCTGCTTC	Construction of H1 strain
prom1_PacI_FWD	CCGTTGCGGGCTTTTCCCGTTTGACACGGGGGCGGGAGGTTAATTAAATTCCGGGGATCCGTCGACC	*topA*p1 disruption
prom1_PacI_RV	CCCGGGGTCGGGTCGTTCTGTGTTTCGATCGTGACGCTGTTAATTAATGTAGGCTGGAGCTGCTTC	*topA*p1 disruption
prom2_PacI_FWD	CGGAGCGGGTCAGCACTCCGGGCGGCAGGAACGGGGTTTTAATTAAATTCCGGGGATCCGTCGACC	*topA*p2 disruption
prom2_PacI_RV	CCCCCGTGTCAAACGGGAAAAGCCCGCAACGGCCACTCGTTAATTAATGTAGGCTGGAGCTGCTTC	*topA*p2 disruption
*3543*_400FW	GTGGACCACGACGCGGGTGC	translation start mapping
*3543*_77RV	GATCGTCTTCGCCTTGGCGGG	translation start mapping
M13FW	GTAAAACGACGGCCAG	translation start mapping
blap1apraFW	CCCTGATAAATGCTTCAATAATATTGAAAAAGGAAGAGTATAACTTATGAGCTCAGCCAATC	*amp*^{R} gene replacement
balp2apraRV	AATCTAAAGTATATATGAGTAAACTTGGTCTGACAGTTAAGTTCCCGCCAGCCTCGCAGA	*amp*^{R} gene replacement
pForHyg6902	GTGCCGTTGATCGTGCTATG	*apra*^{R} gene replacement
pRevHyg6902	CCTTGCCCCTCCAACGTCATCTCGTTCTCCGCTCATGAGCTCAGGCGCCGGGGGCGG	*apra*^{R} gene replacement
*topA*promBamHIFW	GCGGATCCGATCTTCGCGGGAGTGGTGTTC	pFLUXH cloning
*topA*promNdeIRV	CGCATATGCGCTCTTCTCTCCGGTCGACGC	pFLUXH cloning
prom*3543*RTPCRFW	GATCGAAACACAGAACGACC	RT-qPCR/promoter mapping
prom_p2_*3543*_FW	CCGTTGCGGGCTTTTCCCG	RT-qPCR/promoter mapping
prom3_*topA*_FW	GGGTTCGTCGGGTTACCG	promoter mapping

Table S2. Characterization of *S. coelicolor* strains under different supercoiling conditions

Strain [1]	Inducer/antibiotic	TopA level[2]	*topA*p1 specific transcript[3]	*topA* overall transcript	*gyrB* overall transcript	Reporter plasmid[4]
M145 (MS10)	none	1.00	1.00± 0.15	1.00[2] ± 0.11	1.00[2] ± 0.04	-0.030<br>See Fig. 5 and Fig. 3
PS04 (MS11)	none	below detection	2.47[3] ± 0.20	0.05 ± 0.01	0.84 ± 0.11	-0.037<br>See Fig. 3
PS04 (MS11)	Thiostrepton<br>2.0 µg/ml	0.64	nd	1.31 ± 0.10	1.00 ± 0.05	nd
PS04 (MS11)	Thiostrepton<br>5.0 µg/ml	1.25	nd	7.44 ± 0.35	1.86 ± 0.27	-0.033[5]<br>See Fig. S4
PS07 (MS12)	none	0.86	1.14 ± 0.08	1.28 ± 0.08	nd	-0.031<br>See Fig. S4
PS07 (MS12)	Thiostrepton<br>5.0 µg/ml	3,44	0.91 ± 0.02	19.52 ± 0.21	nd	-0.024[4]<br>See Fig. S4
M145 (MS10)	Novobiocin<br>1.0 µg/ml	1.01	0.87 ± 0.17	0.87 ± 0.11	2.33 ± 0.27	nd
M145 (MS10)	Novobiocin<br>5.0 µg/ml	0.91	0.83 ± 0.10	0.80 ± 0.13	8.34 ± 1.10	-0.018[6]<br>See Fig. 5

[1] in brackets the corresponding strain carrying the reporter plasmid

[2] the data were related to the wild type strain (M145) equated to 1.00

[3] the transcript specific to *topA*p1 promoter was determined in the MG04 strain (PS04, with the reporter genes replacing *topA* in the native locus, under control of the *topA* promoter)

[4] the median value of superhelical density was calculated using Fiji Software

[5] the value calculated 60 minutes after exposition to thiostrepton

[6] the value calculated 10 minutes after exposition to novobiocin

Figure S1

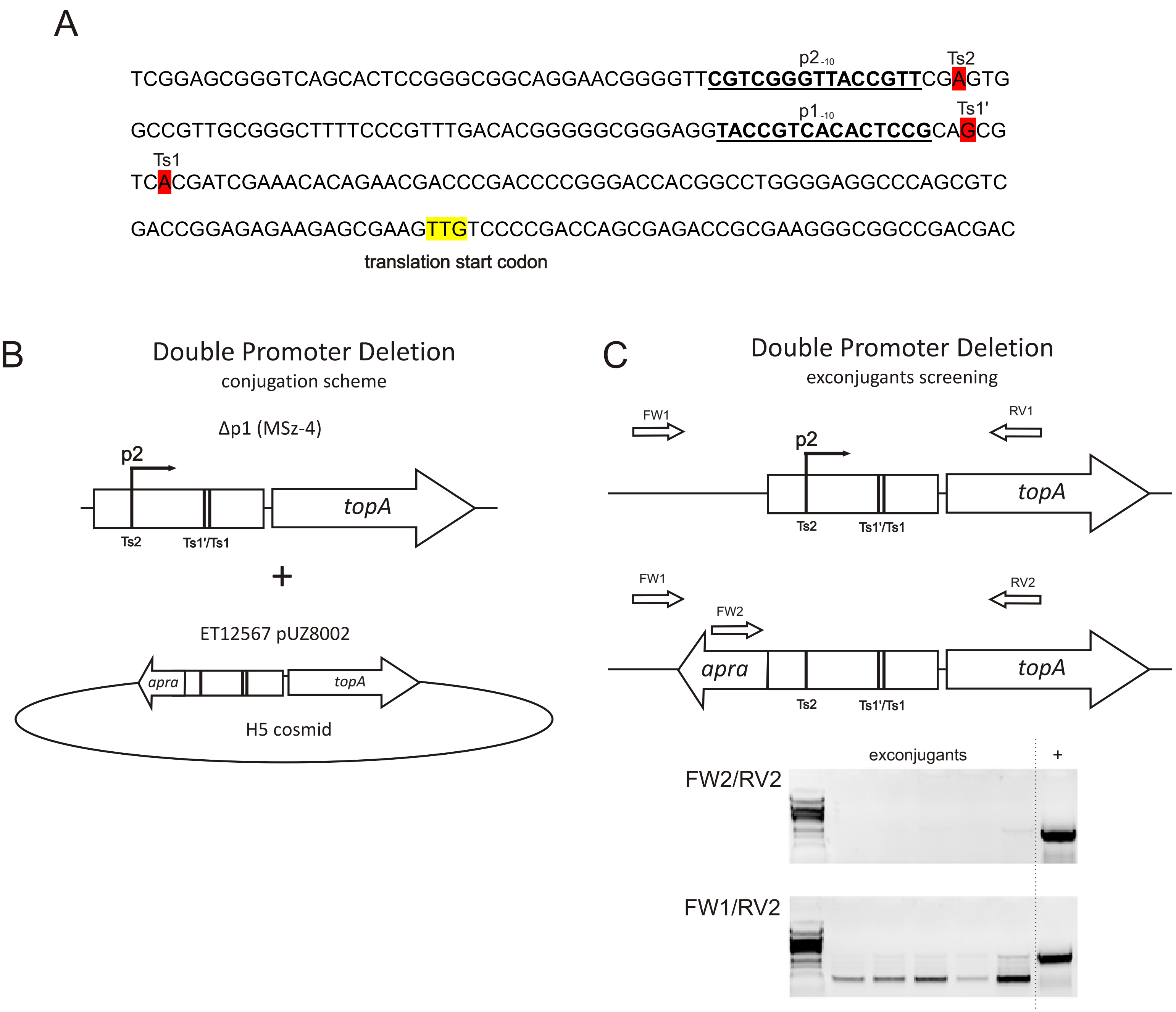

Figure S1. Construction of p1 and/or p2 mutants of the *S. coelicolor topA* promoter. (**A**) To disrupt p1 (MSz-4) or p2 (MSz-1a) promoter of the *topA* gene the seqenece (underlined) upstream transcription start point (red) was replaced with TTAATTAA seqence recognized by PacI restriction enzyme. For more details see *Materials and Metods.* (**B**) To disrupt the p1 and p2 promoters, we used the MSz-4 strain conjugated to the H5 cosmid carrying a double p1/p2 mutation. Screening the double exconjugants with specific oligonucleotides. (**C**) Double promoter deletion resulted in the introduction of an apramycin resistance cassette in place of the p2 promoter and was detected using FW2/RV2 oligonucleotides (control H5 cosmid - marked with +). The proper recombination pattern was not detected in any of the tested exconjugants. PCR analysis with FW1/RV1 oligonucleotides shows that the promoter region remained intact.

Figure S2

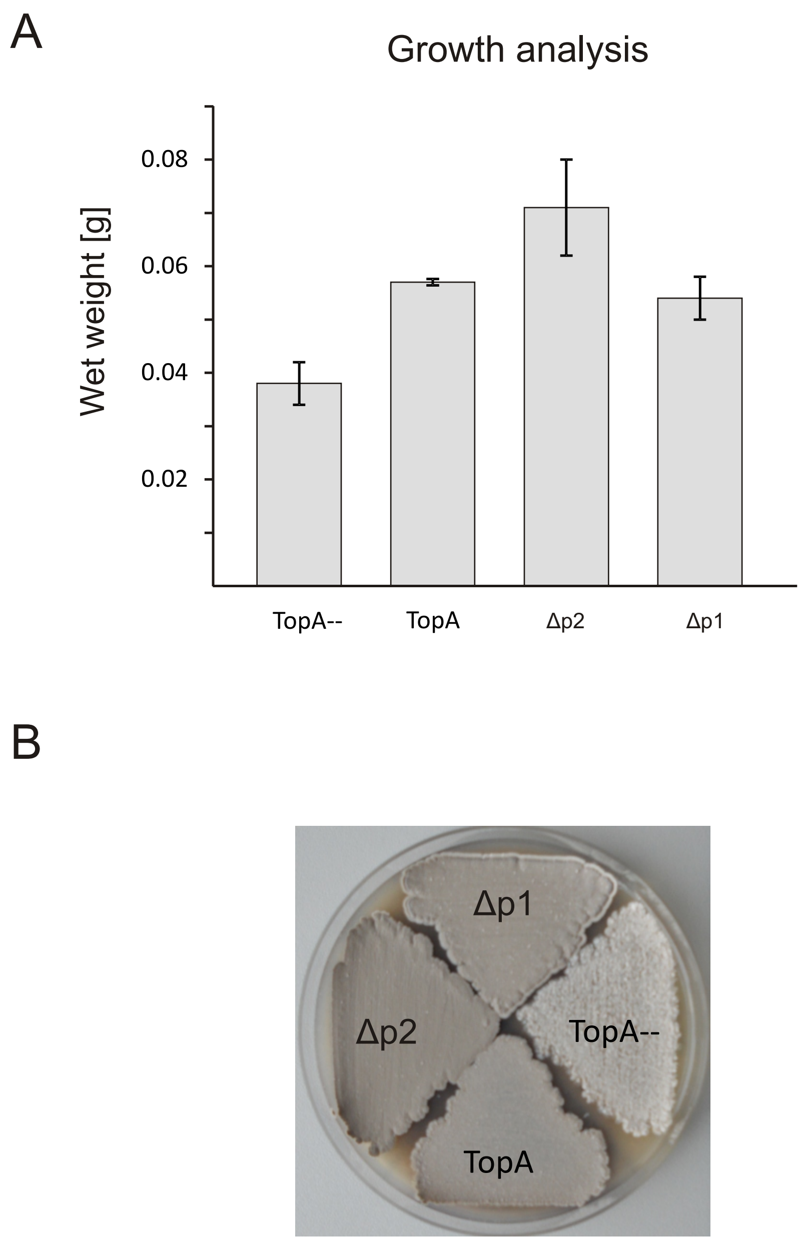

Figure S2. Analysis of the growth and differentiation of *S. coelicolor topA* promoter mutants. (**A**) The growth rate of *S. coelicolor topA* promoter mutants (Δp1 and Δp2), quantified by weighing the cell pellet collected after 24 hours of growth in liquid 79 medium. The wild type strain (TopA) and TopA-depletion strain (TopA-) served as control samples. (**B**) The sporulation of the *topA* promoter mutants (Δp1 and Δp2) was examined on solid MS medium. The wild type strain (TopA, sporulating) and TopA-depletion (TopA-, nonsporulating, white) strains served as controls.

Figure S3

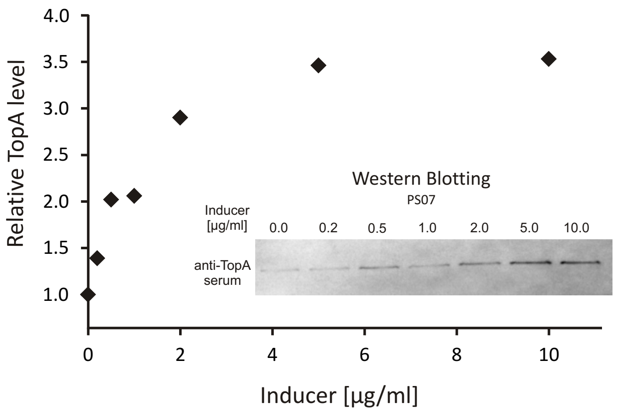

Figure S3. Analysis of TopA level in the PS07 strain growing constantly in the presence of increasing amounts of the inducer, as assessed by western blot with anti-TopA polyclonal antibodies.

Figure S4

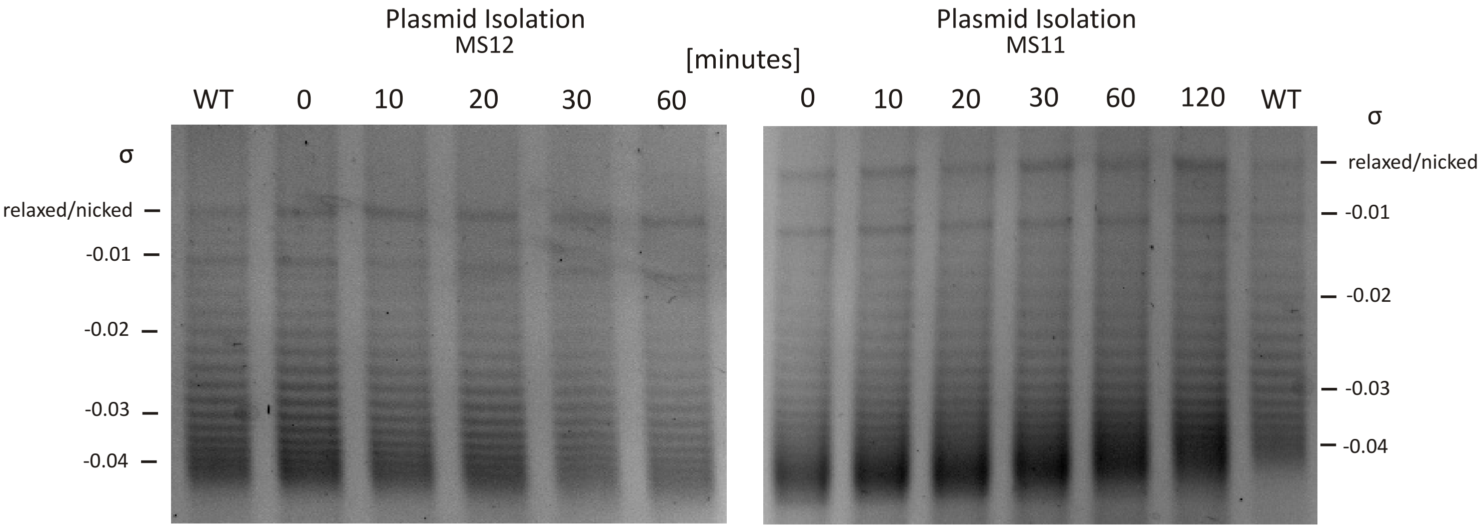

Figure S4. The isolation of the reporter plasmid pWHM3Hyg (**A**) The plasmid isolated from MS12 (a derivative of TopA overproducing PS07 strain) cultured in the presence of the inducer (5µg/ml) in time up to 60 minutes (**B**) The plasmid isolated from MS11 (a derivative of TopA-depleted PS04 strain) cultured in the presence of 5 µg/ml of thiostrepton in time up to 120 minutes. Both strains were compared to the plasmid isolated from the wild type strain not exposed to thiostrepton.

Figure S5

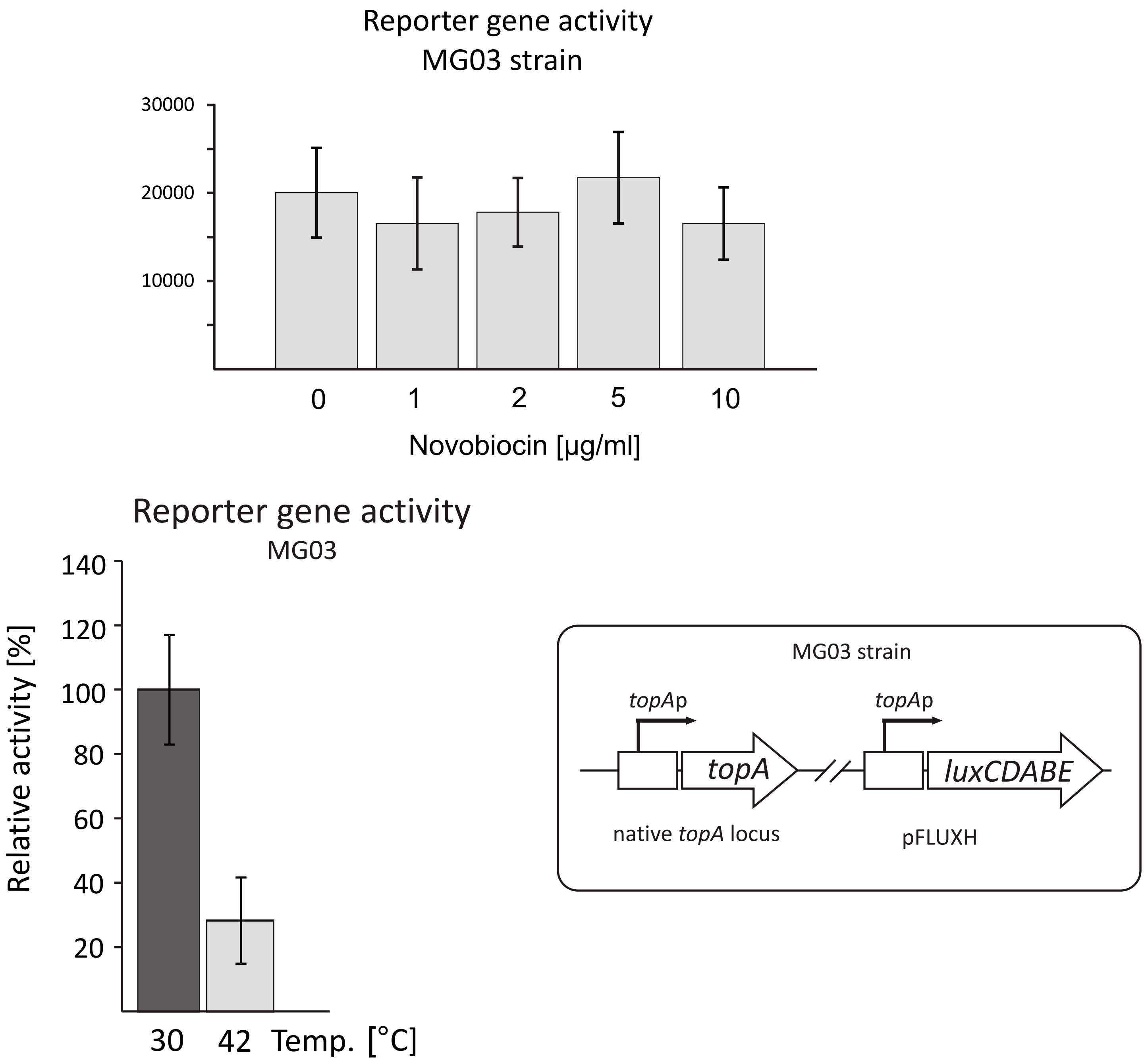

Figure S5. The influence of novobiocin and heat shock on the expression of the reporter gene under control of *topA* promoter in a wild type background. (A) The luciferase activity in the MG03 strain (see the scheme below) after treatment with increasing concentrations of novobiocin. (B) The luciferase activity in the MG03 strain after exposure to 42°C (120 minutes) normalized to standard conditions (30°C).

Figure S6

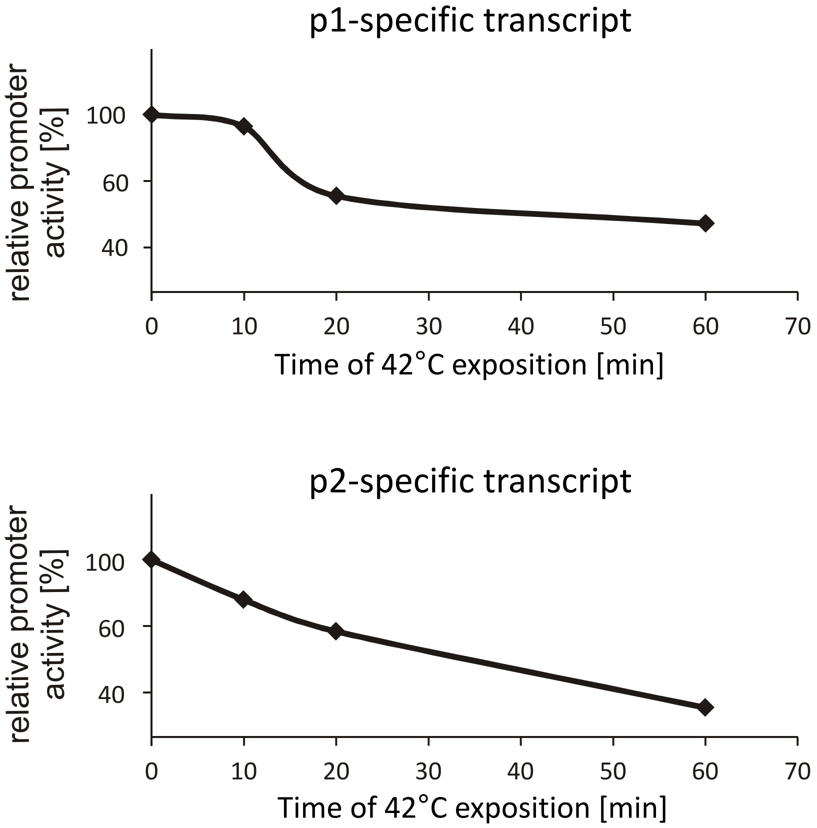

Figure S6. The specific activty of the *topA* p1 and p2 promoters under heat shock conditions. The level of each transcript was quantified using RT-qPCR. The transcript level isolated from cells not exposed to 42°C was estimated as 100%.

Figure S7

A

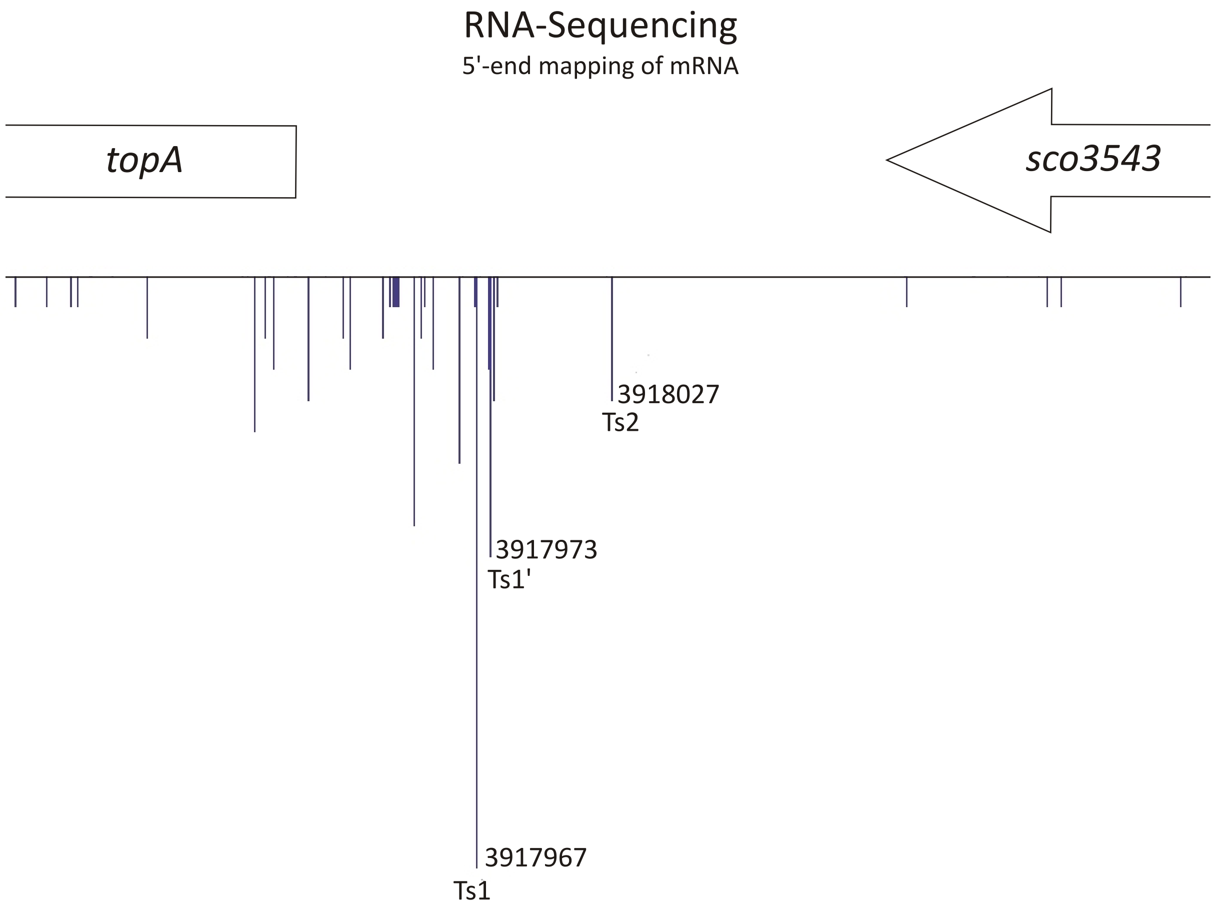

B

	Chromosome position	
**Site**	**S1 mapping**	**RNA-Sequencing**
Ts1	3917968	3917967
Ts1'	3917973	3917973
Ts2	3918029	3918027

Figure S7. Identification of the *topA* transcription start point using 5'-end differential mapping. (**A**) A magnified view of the *topA* upstream region showing the location of transcriptional start points (Ts1/Ts1' and Ts2). The number above corresponds to the chromosomal locus of each site. (**B**) A comparison of the chromosomal loci of the Ts1/Ts1' and Ts2 sites using S1 nuclease mapping and 5'-end mapping.

Figure S8

A

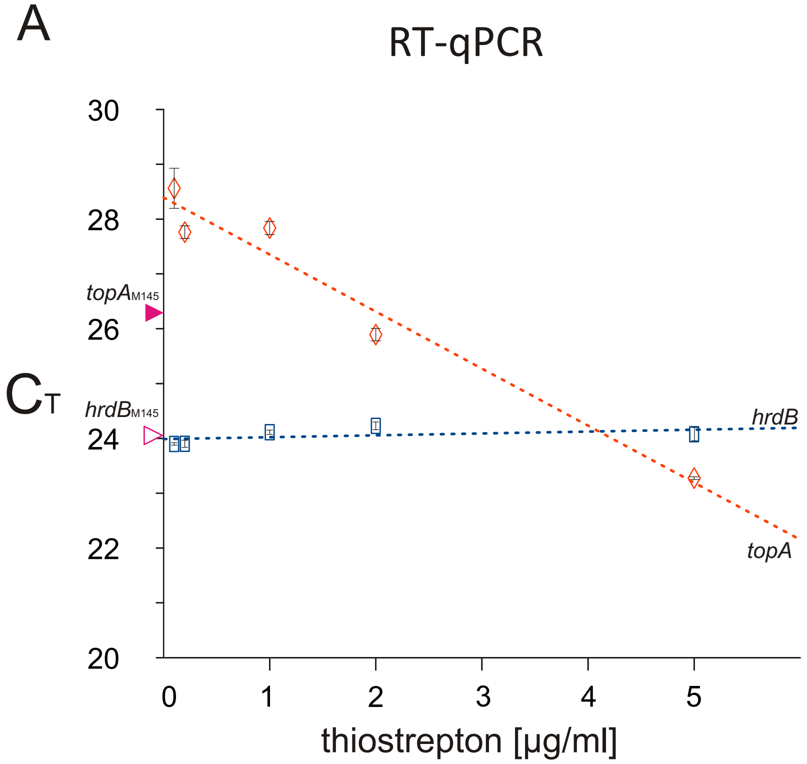

B

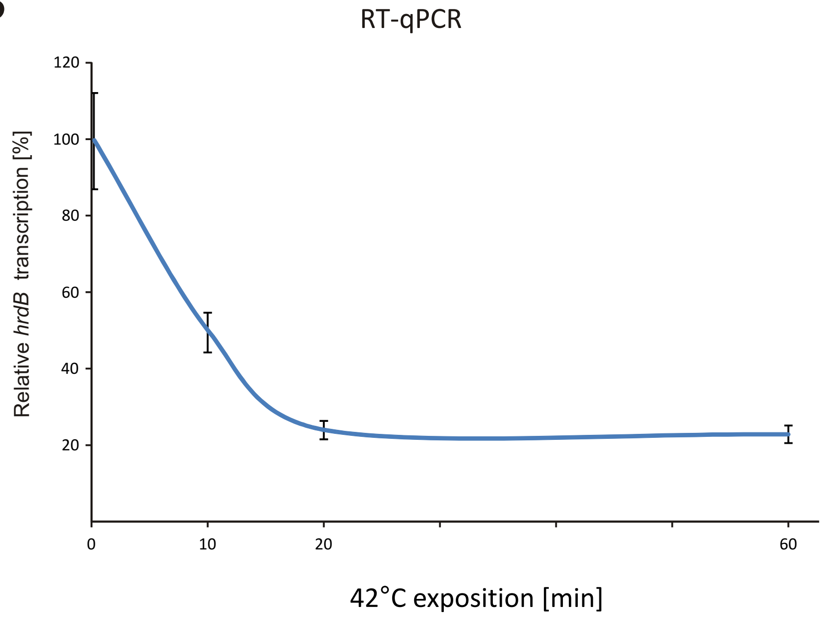

Figure S8. RT-qPCR analysis of *hrdB* gene transcription in *S. coelicolor*. (**A**) A comparison of Ct values for *hrdB* (blue) and *topA* (orange) transcripts. RNA was isolated from PS04 strains with different DNA supercoiling levels. The Ct values corresponding to the wild type level of transcript for *hrdB* (empty arrow) and *topA* (filled arrow) are marked. (**B**) Relative *hrdB* transcription after exposure of the wild type strain to temperature stress (42°C).

Figure S9

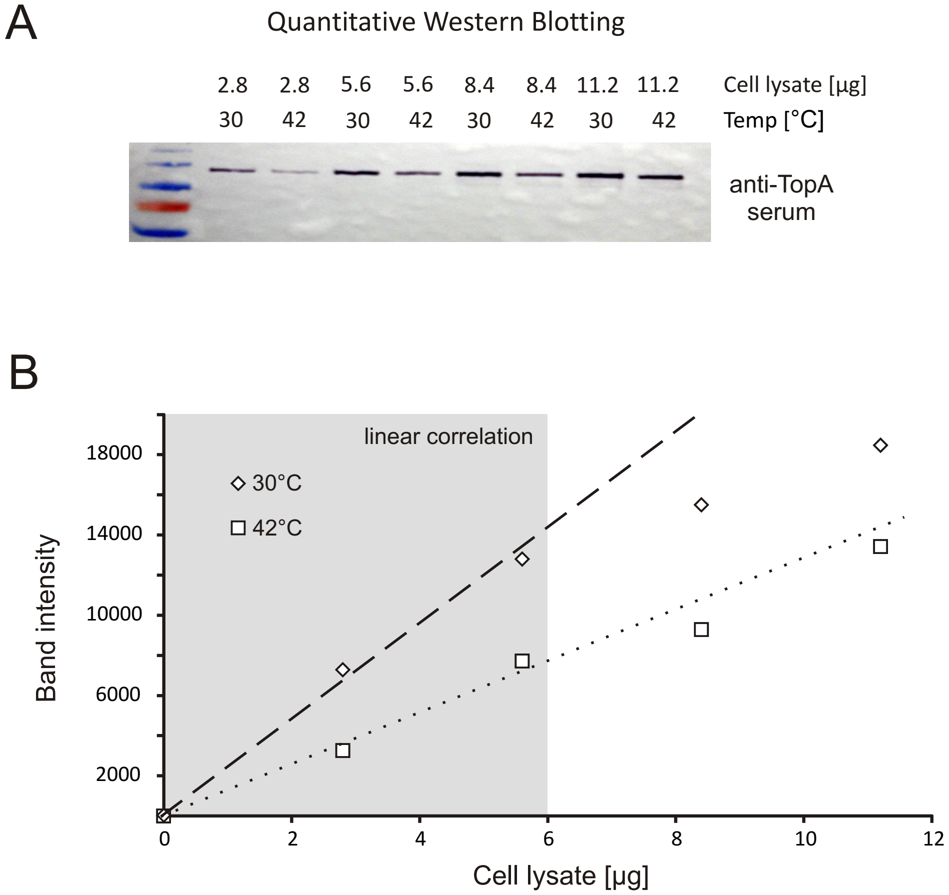

Figure S9. Quantitative western blotting. (**A**) The detection of TopA protein in *S. coelicolor* cell lysate using anti-TopA polyclonal antibody. Prior to disruption, the cells were exposed to an elevated temperature (42°C) for 60 min. Cells that had not been heat-shocked served as a control. Different amounts of cell lysate (µg of total protein) were loaded on the gel, as indicated. (**B**) The correlation of band intensity and the amount of total protein loaded for quantitative Western blotting (the range of linear correlation is marked in grey). The band intensity was measured using ImageJ2x software.

MIQE form. RT-qPCR and sample information

Experimental Design

Definition of experimental and control groups	E	*Streptomyces coelicolor* at different stages of culture and/or culture exposed to high temperature
Number within each group	E	Each sample was repeated in 1-3 biological replicates
Assay carried out by the core or investigator's laboratory	D	Assay carried out by investigator lab
Acknowledgment of authors' contribution	D	-

Sample

Description	E	*Streptomyces coelicolor* growing for 24 hours (180 rpm shaking, 37ºC) in liquid 79 medium. To induce heat-shock cell were exposed to 42ºC for 10-120 minutes
Volume/mass of sample processed	D	50-100 mg of vegetative hyphae after 24 hours of growth
Microdissection or macrodissection	E	-
Processing procedure	E	Collected hyphae was washed with 5ml of cold PBS buffer and immediately processed using manufacturers protocol (see also section: Name of kit and details of any modifications)
If frozen, how and how quickly?	E	-
If fixed, with what and how quickly?	E	-
Sample storage conditions and duration (especially for FFPE[2] samples)	E	-20ºC until use

Nucleic acid extraction

Procedure and/or instrumentation	E	Total RNA from *S. coelicolor* vegetative hyphae was isolated using modified manufacturers protocol (see details below)

Name of kit and details of any modifications	E	Total RNA was isolated using GeneJet RNA Isolation Kit (Thermo Scientific) usinge the modified manufacturers protocol (Bacterila Total RNA purification procedure D) #1 step 2: before lysis, cell were treated with 200 µl of lysozyme solution (1mg/ml); #2 step 3: 4-5 drops of zirconia beads in PBS buffer were added and vortexed for 1 min to increases cell disruption efficiency; #3 step 4 and step 5: the volumes of lysis buffer and 100% ethanol were doubled
Source of additional reagents used	D	-
Details of DNase or RNase treatment	E	DNA traces were removed by treatment of 2 µg of isolated RNA with RNase-free DNase I (Thremo Scientific) following manufacturers instruction
Contamination assessment (DNA or RNA)	E	RNA after DNase I treatment served as template for standard PCR reaction with RT3543RV and prom3543RTPCR_FW oligonucleotides. No detectable product after 30 reaction cycles proved elimination of DNA traces (chromosomal DNA)
Nucleic acid quantification	E	The RNA quality was tested using standard gel electrophoresis in the presence of ethidium bromide. Only RNA samples with no detectable degradation was used for cDNA synthesis. The RNA quantity was measured spectrophotometrically
Instrument and method	E	NanoDrop 1000 Spectrophotometer
Purity (A_{260}/A_{280})	D	samples with OD ratio of at least 1,8 were used for further analysis
Yield	D	-
RNA integrity	E	agarose-gel electrophoresis
RIN/RQI or Cq of 3’ and 5’ transcripts	E	not performed
Electrophoresis traces	E	not performed
Inhibition testing (Cq dilutions, spike, or other)	E	not performed

Reverse transcription

Complete reaction conditions	E	cDNA was synthesized from 1 µg of total RNA using iScript Reverse Transcription Kit (Bio-Rad) following manufacturer instructions
Amount of RNA and reaction volume	E	1 µg in 20 µl
Priming oligonucleotides (if using GSP) and concentrations	E	random primers, according to the manufacturer instructions
Reverse transcriptase and concentration	E	according to the manufacturer instructions
Temperature and time	E	according to the manufacturer instructions
Manufacturer of reagents and catalogue numbers	D	Bio-Rad
C_qs with and without reverse transcription	D	not performed
Storage conditions of cDNA	D	-20ºC

qPCR target information

Gene symbol	E	see primer and amplicon information on this file
Sequence accession number	E	see primer and amplicon information on this file
Location of amplicon	D	-
Amplicon length	E	see primer and amplicon information on this file
In silico specificity screen (BLAST, and so on)	E	analysis not performed
Pseudogenes, retropseudogenes, or other		

homologs?	D	-
Secondary structure analysis of amplicon	D	analysis not performed
Location of each primer by exon or intron	E	not applicable
What splice variants are targeted?	E	not applicable

qPCR oligonucleotides

Primer sequences	E	see primer and amplicon information on this file
RTPrimerDB identification number	D	-
Probe sequences	D	not applicable
Location and identity of any modifications	E	none
Manufacturer of oligonucleotide	D	Sigma Aldrich
Purification method	D	none

qPCR protocol

Complete reaction conditions	E	20 µl reaction contained forward and reverse oligonucleotides in concentration 20 pmol/µl, 10 µl of SYBR Green Master Mix, 120 ng of cDNA
Reaction volume and amount of cDNA/DNA	E	See details above
Primer, (probe), Mg^{2+}, and dNTP concentrations	E	See detail above, Mg^{2+} and dNTP were supplied in SYBR Green Master Mix

Polymerase identity and concentration	E	according to the manufacturer instructions
Buffer/kit identity and manufacturer	E	iTaq Universal SYBR Green Supermix (Bio-Rad)
Exact chemical composition of the buffer	D	-
Additives (SYBR Green I, DMSO, and so forth)	E	-
Manufacturer of plates/tubes and catalog number	D	Applied Biosystems
Complete thermocycling parameters	E	10 min at 95ºC, followed by 40 cycles of 15 s at 95ºC, 15 s at 58ºC, and 15 s 72ºC. The amplification process was followed by a melting curve analysis
Reaction setup (manual/robotic)	D	-
Manufacturer of qPCR instrument	E	StepOnePlus Real-Time PCR System (Applied Biosystems)
**qPCR validation**		
Evidence of optimization (from gradients)	D	-
Specificity (gel, sequence, melt, or digest)	E	Specifity was validated with melt curve analysis (see information on this file)
For SYBR Green I, Cq of the NTC	E	Ct undetected (see information on this file)
Calibration curves with slope and y intercept	E	see information on this file
PCR efficiency calculated from slope	E	see information on this file
CIs for PCR efficiency or SE	D	-
r2 of calibration curve	E	see information on this file
Linear dynamic range	E	Ct20 to Ct35
Cq variation at LOD	E	-

CIs throughout range	D	-
Evidence for LOD	E	-
If multiplex, efficiency and LOD of each assay	E	-

Data analysis

qPCR analysis program (source, version)	E	StepOne Software Version 2.3 (Life Technologies)
Method of Cq determination	E	The fluorescence threshold for determining Cq values is automatically set at 1 cycle below the upper limit of the window of linearity
Outlier identification and disposition	E	-
Results for NTCs	E	Ct undetected, see information on this file and section: Contamination assessment (DNA or RNA)
Justification of number and choice of reference genes	E	*hrdB*, see information on this file
Description of normalization method	E	The copy number of each transcript was quantified using calibration curve prepared for H5 cosmid (carrying the *S. coelicolor* chromosome fragment with targeted gene). For some experiments (see detail in the manuscript) normalized gene expression ratios were calculated by the ΔΔCt method with hrdB as a reference gene.
Number and concordance of biological replicated	D	-
Number and stage (reverse transcription or qPCR) of technical replicates	E	three
Repeatability (intraassay variation)	E	see figures attached to the manuscript
Reproducibility (interassay variation, CV)	D	-

Power analysis	D	-
Statistical methods for results significance	E	-
Software (source, version)	E	algorithms incorporated to the StepOne Software Version 2.3
Cq or raw data submission with RDML	D	-

MIQE form. The oligonucleotides used in qPCR experiments

**Oligonucleotide**	**Sequence**
*hrdB*RT_f	TGCTCTTCCTGGACCTCATC
*hrdB*RT_r	GTAGCCCTTGGTGTAGTCGAA
RT*3543*FWD	ACGACTTCCAGCCGATCTATGT
RT*3543*RV	GGAACACCATGCGCTTGAC
prom_p2_*3543*_FW	CCGTTGCGGGCTTTTCCCG
prom2_166RV	CTTCGCGGTCTCGCTGGTC
RTPCR*3874*RV	TCGAGGGACAGGTCTTGTC
RTPCR*3874*FW	TCGACTTCGTGACGTACCTCAA
*luxC*_FW	CGGCGACAACAGCGTCTA
*luxC*_RV	CAACCGCAGCTTGTTGTTCTC
prom_p1_*3543*_FW	GATCGAAACACAGAACGACC
prom1_170_RV	GGCCGAGATAGCCCTTGATC

MIQE form. The amplicon details

Oligonucleotides	Amplicon name	Amplicon length	Tm [ºC]	Slope	Efficiency	r^2
RT*3543*FWD/ RT*3543*RV	P1+P2	186 bp	84.87	-3.581	90.221%	0.998
prom_p2_*3543*_FW/ prom2_166RV	P2	166 bp	87.69	-3.673	87.176%	0.993
prom_p1_3543_FW/ prom1_170_RV	P1	170 bp	85.87	-3.788	83.656%	0.986
*luxC*_FW/*luxC*_RV	P1+P2_lux	88 bp	80.65	-3.493	93.343%	0.994
prom_p2_*3543*_FW/ *luxC*_RV	P2_lux	310 bp	86.75	-3.512	92.636%	0.999
*hrdB*RT_f/*hrdB*RT_r	*hrdB*	82 bp	82.35	nd	nd	nd
RTPCR*3874*FW/RTPCR*3874*RV	*gyrase*	100 bp	83.99	nd	nd	nd
